# *Taenia solium* and *Taenia crassiceps*: miRNomes of the larvae and effects of miR-10-5p and let-7-5p on murine peritoneal macrophages

**DOI:** 10.1042/BSR20190152

**Published:** 2019-11-19

**Authors:** Abraham Landa, Luz Navarro, Alicia Ochoa-Sánchez, Lucía Jiménez

**Affiliations:** 1 Departamento de Microbiología y Parasitología, Facultad de Medicina, Universidad Nacional Autónoma de México, CDMX 04510, Mexico; 2 Departamento de Fisiología, Facultad de Medicina, Universidad Nacional Autónoma de México, CDMX 04510, México

**Keywords:** Gene regulation, Larvae, Macrophages, microRNA (miRs), Taenia solium

## Abstract

Neurocysticercosis (NCC), a major cause of neurological morbidity worldwide, is caused by the larvae of *Taenia solium*. Cestodes secrete molecules that block the Th1 response of their hosts and induce a Th2 response permissive to their establishment. Mature microRNAs (miRs) are small noncoding RNAs that regulate gene expression and participate in immunological processes. To determine the participation of *Taenia* miRs in the immune response against cysticercosis, we constructed small RNA (sRNA) libraries from larvae of *Taenia solium* and *Taenia crassiceps*. A total of 12074504 and 11779456 sequencing reads for *T. solium* and *T. crassiceps*, respectively, were mapped to the genomes of *T. solium* and other helminths. Both larvae shared similar miRNome, and miR-10-5p was the most abundant in both species, followed by let-7-5p in *T. solium* and miR-4989-3p in *T. crassiceps*, whereas among the genus-specific miRs, miR-001-3p was the most abundant in both, followed by miR-002-3p in *T. solium* and miR-003a-3p in *T. crassiceps*. The sequences of these miRs were identical in both. Structure and target prediction analyses revealed that these pre-miRs formed a hairpin and had more than one target involved in immunoregulation. Culture of macrophages, RT-PCR and ELISA assays showed that cells internalized miR-10-5p and let-7-5p into the cytoplasm and the miRs strongly decreased interleukin 16 (*Il6*) expression, tumor necrosis factor (TNF) and IL-12 secretion, and moderately decreased nitric oxide synthase inducible (*Nos2*) and *Il1b* expression (pro-inflammatory cytokines) in M(IFN-γ) macrophages and expression of *Tgf1b*, and the secretion of IL-10 (anti-inflammatory cytokines) in M(IL-4) macrophages. These findings could help us understand the role of miRs in the host–*Taenia* relationship.

## Introduction

Neurocysticercosis (NCC) in humans is caused by larvae of *Taenia solium*. It is the leading cause of seizures and epilepsy and is considered a serious health problem worldwide [1]. In human NCC, the intensity of symptoms depends primarily on the inflammatory response, which is associated with the Th1 response with high levels of TNF, IFN-γ, IL-17, and IL-23 and coincides with degeneration of the larvae [2,3], whereas the Th2 response (anti- inflammatory response) is associated with asymptomatic NCC with high level of IL-10, IL- 4, IL5, and IL-13 and viable larvae [3,4]. In animal NCC, swine and murine granulomas and their inflammatory responses are similar than in humans [5–8]. However, the exact role of classically and alternatively activated macrophages/microglia and their secreted molecules in NCC patients remains to be elucidated. In contrast, in the model of murine peritoneal cysticercosis by *Taenia crassiceps*, the macrophages play a key role in promoting the Th1 (protective) and Th2 (permissive) responses against the larvae [9,10]. The depletion of alternatively activated macrophages during early infection decreases the parasitic burden and restores the antigen-specific proliferative response in T lymphocytes [11,12]. It is also known that the products of *T. crassiceps* larvae block the toll-like receptor (TLR) response and the production of the inflammatory cytokines in both macrophages and dendritic cells from mice, biasing to Th2 environment that includes T cells (Th2 and Treg), eosinophils, and alternatively activated macrophages with the secretion of type 2 cytokines that facilitate larval growth [13–15].

Mature microRNAs (miRs) are small noncoding RNAs (∼22 nt) that bind to complementary sequences in the 3′ untranslated region (3′-UTR) of target mRNAs and mediate either translational repression or mRNA degradation [16,17]. They are involved in processes, such as developmental transitions, responses to the environment, cell signaling, and in the regulation of the immune response [16–18]. In addition, miRs are also associated with M1 and M2 activation in human and murine macrophages [19–22]. In *Taenia* genus, adult-stage miRs have been reported in *T. multiceps, T. saginata, T. asiatica*, and *T. solium*, showing similar profiles; in contrast, only one report of the larval stage miR of *T. ovis* was found [23–26]. However, functional tests for these miRs have not been performed.

The present study aimed to sequence the miRs from the larvae of *T. solium* and *T. crassiceps* to compare their expression profiles, as well as bioinformatics analysis of their structure and abundance and the prediction of their immunological targets. In addition, we show the effects of the two most abundant miRs on naive and activated peritoneal macrophages.

## Materials and methods

### Biological material

*Taenia crassiceps* (ORF strain) larvae were obtained from experimentally infected mice, as previously described [10], and then, killed in a CO_2_ chamber 90 days later. *T. solium* larvae were dissected from naturally infected rural pigs acquired from local farms. Larvae were washed four times with sterile ice-cold phosphate-buffered saline (PBS) pH 7.2 and stored at −70°C. Naive macrophages were obtained from non-infected mice.

## Construction of the small RNA library and sequence analysis

Total RNAs from both *Taenia* larvae were prepared using TRIzol reagent (Invitrogen, Carlsbad, CA, U.S.A.) as described before [27] and the small RNA (sRNA) fractions were obtained with the protocol of the mirVana™ miRNA Isolation Kit (Life Technologies). Concentration and purity were analyzed using the Agilent 2100 and RNA 6000 Nano LabChip Kit (Santa Clara, CA, U.S.A.), respectively. sRNA library was generated using the Illumina Truseq™ Small RNA Preparation kit according to Illumina’s TruSeq™ Small RNA Sample Preparation Guide (Illumina, San Diego, CA, U.S.A.) and single-end sequencing (36 bp) was performed and analyzed on the Illumina HiSeq2500 system and Illumina’s Sequencing Control Studio software (version 2.8) (LC Sciences Hangzhou, China), respectively, according to the manufacturer’s instructions. Unmappable reads and reads <13 or >32 bases were removed. Data were used to map the reference files from the *T. solium* genome database (ftp://ftp.ebi.ac.uk/pub/databases/wormbase/parasite/releases/WBPS7/species/taenia_solium/PRJNA170813/taeniasolium.PRJNA170813.WBPS7.genomic.fa.gz) and miR and pre-miR database (ftp://mirbase.org/pub/mirbase/). Target prediction and analysis of structure were performed using target sites available on the web (http://mirdb.org; http://www.targetscan.org; and http://rna.tbi.univie.ac.at//cgi-bin/RNAWebSuite/RNAfold.cgi).

## Uptake of miRs by macrophages

Naive murine macrophages were isolated from the peritoneum by aspiration with RPMI and 2 × 10^6^ cells/ml were seeded on sterilized coverslips coated with poly-d-lysine (Neuvitro, Vancouver, WA, U.S.A.) in a culture plate, as previously described [28]. Cultures were incubated in RPMI-1640 with 10% FBS, 100 U/ml penicillin, and 100 μg streptomycin in an atmosphere of 5% CO_2_ at 37°C for 15, 30, or 60 min in the absence or presence of synthetic miR-10-5p or let-7-5p (100 ng/ml) coupled to Cyanine 5 (Cy5) (Sigma, St Louis, MO, U.S.A.). Cells were washed once with PBS and fixed with 4% paraformaldehyde for 1 h at 4°C. The cells were washed three times with PBS, and then, stained with 4′,6-diamidino-2-phenylindole (DAPI) for 5 min. After one more wash with PBS, the macrophages adhered to the coverslips were mounted on to slides using 50% glycerin in PBS. Fluorescence was detected using a laser scanning confocal microscope at the excitation wavelengths of 405 and 603 nm for DAPI and Cy5, respectively. Images were captured under a vertical LEICA TCS-SP5 II microscope and processed with the manufacturer’s software. Three-channel images were acquired in the light field microscopy and fluorescence microscopy to detect DAPI and Cy5, and then, merged for analysis. Orthogonal images were obtained by laser scanning in an XYZ configuration (3D reconstruction), taken in projections of 17–18 sequential sections, scanned five to seven times each, with a thickness of 10.5 μm.

## *In vitro* stimulation of naive peritoneal macrophages with miRs

Groups of naive macrophages (2 × 10^6^/ml) were cultured without treatment (designated M(N)) or activated with 20 ng/ml IL-4 (designated as M(IL-4)) or 100 ng/ml IFN-γ (designated M(IFN-γ)) [29]. Then, macrophages were incubated with synthetic miR-10-5p or let-7-5p (Sigma, St. Louis, MO, U.S.A.) at 1, 10, 100, and 200 nM concentrations. After 24 h, the cells were washed with PBS and lysed to obtain the total RNA, as described above [27]. Determination of the expression of various genes (glyceraldehyde 3-phosphate dehydrogenase (*Gapdh*), nitric oxide synthase inducible (*Nos2*), interleukin 6 (*Il6*), transforming growth factor β1 (*Tgb1*), and interleukin 1β (*Il1b*)) was performed by RT-PCR with the SuperScript One-Step RT-PCR Kit (Invitrogen, Carlsbad, CA, U.S.A.), using 1 μg of total RNA and primers described previously [30–32]. The *Gapdh* expression was used to normalize the band densities of the other gene transcripts. Detection of IL-10, TNF, and IL-12 cytokine levels in the culture supernatant was carried out using the corresponding Murine Standard ABTS ELISA Development Kit (Peprotech, Rocky Hill, NJ, U.S.A.) following the manufacturer’s instructions.

## Statistical analysis

One-way Analysis of Variance (ANOVA) with Tukey’s multiple comparisons test was used for the statistical analysis of collected data and *P*-values <0.05 were considered significant. We performed all possible comparisons, however only those comparisons relevant to the discussion are described in the result: to macrophages M(N) vs. M(IFNγ), M(N) vs. M(IL-4), No incubation vs. miR-10-5p, and No incubation vs. let-7-5p.

## Results

### Isolation of sRNAs from *T. solium* and *T. crassiceps* larvae

**Figure 1:**
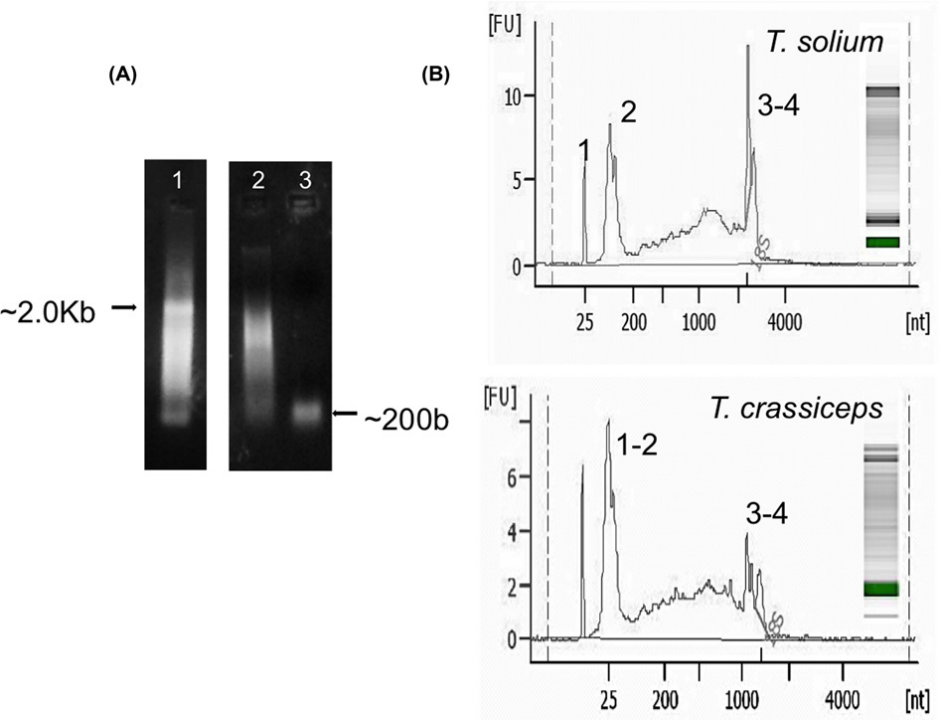
Purification of small RNA (sRNA) (**A**) Agarose gel showing sRNAs purified from *T. solium* larvae. Lane 1: total RNA, lane 2: RNA fraction of >200 nts, and lane 3 sRNA fraction of ∼200 nts, assessed using the miRVana kit. (**B**) Electropherogram plots showing the size (nt) and composition of *T. solium* (Up) and *T. crassiceps* (Down) total RNAs, determined by fluorescence intensity (FU) with the Bioanalyzer 2100 system.

Total RNA extracted from *T. solium and T. crassiceps* larvae presented the characteristic pattern of the RNAs from cestodes (Figure 1A, lane 1), showing the majority of mRNAs between 0.5 and ∼7000 nt, a single ribosomal RNA (rRNA) band of ∼2438 nts (composed of 18S and 28S rRNAs), and an sRNA fraction of ∼200 nts. The RNA fraction of >200 nts (lane 2) and the sRNA fraction (lane 3) were isolated. Figure 1B shows the electropherogram plots of the fluorescence intensities of the bands that form the *T. solium* (Up) and *T. crassiceps* (Down) total RNA, with a peak of ∼25 nts (peak 1) corresponding to putative miRs, a peak of ∼100 nts (peak 2) corresponding to other sRNAs (transfer RNA (tRNA), small interfering RNA (siRNA), and small nucleolar RNA (snoRNA)), and peaks between 2000 and 2500 nts (peaks 3 and 4) corresponding to rRNAs and larger RNAs.

### Characterization of the sRNA libraries

**Figure 2:**
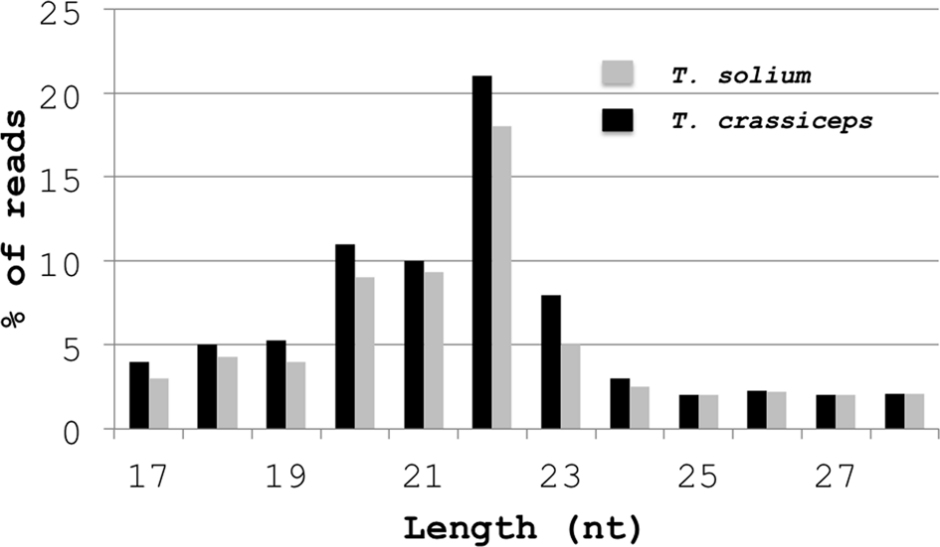
Histogram representing the length distribution of mappable reads in the sRNA libraries of larvae from *T. solium* (gray) and *T. crassiceps* (black) Reads were obtained after removing the 3′-adapter.

The sequences of sRNA libraries showed a total of 12074504 raw reads obtained for *T. solium*, of which 8300154 reads were mappable. For *T. crassiceps*, a total of 11779456 reads were obtained, of which 8256769 were mappable. The mappable reads from *T. solium* (58.2%) and *T. crassiceps* (50.7%), correspond to know and predicted miRs, 12.8 and 10.2%, respectively were mapped to mRNAs, and 14.2 and 11.4%, respectively, were mapped to snoRNAs, siRNA, tRNAs, and rRNA fragments. We found 19.4% reads with no hits to *T. solium* sequence data, and 32% reads with no hits to *T. crassiceps* sequence data. Table 1 shows the known and predicted miRs grouped as A, B, C, D, and E, as well as the number of unique miRs and corresponding reads. Notably, a higher number of unique miRs belonged to the group of predicted miRs mapped to the genome of *T. solium*, but not to that of other helminths (Group D). Moreover, we found 41 unique known miRs in *T. solium* and 40 in *T. crassiceps* that were mappable to the genomes of *T. solium* and other helminths. The total numbers of unique miRs were 335 in *T. solium* and 192 in *T. crassiceps*. For the known *T. solium* miRs, 48.8% were detected at the 5p arm and 51.22% at the 3p arm in 93 clusters of which 67.74% showed reads with zero errors and a score of >500. For the known *T. crassiceps* miRs, 47.73% were detected at the 5p arm and 52.27% at the 3p arm in 99 clusters, of which 64.65% showed reads with zero errors. The GC contents in these miR sequences were between 50 and 70%, and an sRNA population of between 17 and 28 nts (with a predominant size at 22 nts) was observed in both species (Figure 2).

**Table 1:** Unique miRs and their corresponding number of reads found in the sRNA libraries from *T. solium* and *T. crassiceps* cysticerci

miRs group	Reads *T. crassiceps*	Unique miRs	Reads *T. solium*	Unique miRs
A **Known**	2287968	40	1953918	41
B **Predicted, within hairpins**	153574	19	156055	20
C **Predicted, no hairpins**	10159	10	17994	12
D **Predicted unknown *Taenia* genus**	69067	123	93097	262
E **Other**	4065808	NM	3463454	NM
**Total**		192		335

Group A: mapped to helminth genomes in miRbase; Groups B and C: mapped to helminth and *T. solium* genomes; Group D: mapped only to the *T. solium* genome; and Group E: corresponding to reads mapped to mRNA, Rfam or rep databases (NM = no miRs).

### Characterization of the known and specific unknown miRs from the larvae

**Figure 3:**
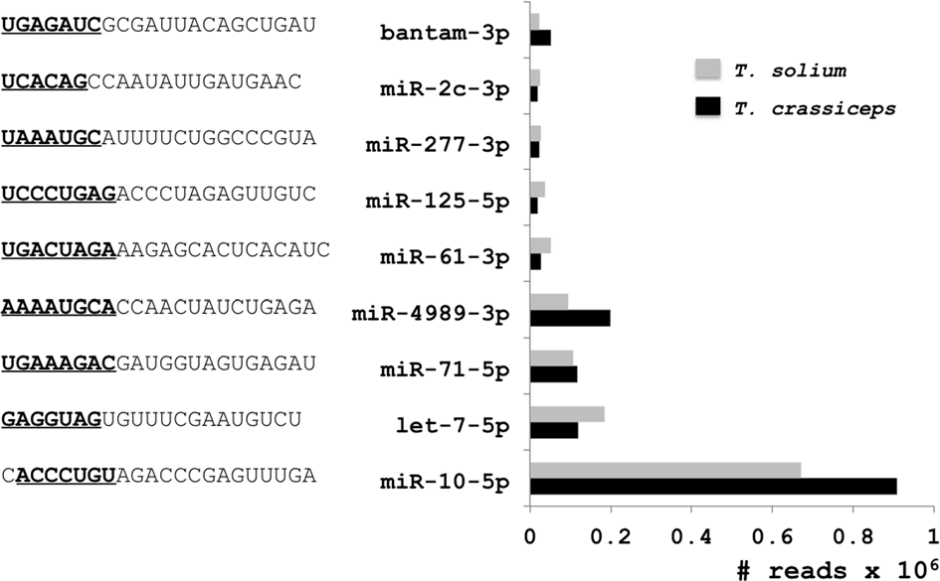
Sequences and number of reads of the most abundant mature miRs in the larvae of *T. solium* (gray) and *T. crassiceps* (black) The cutoff for the number of copies was 20000 reads. The references were the miRbase, *Schistosoma* spp., *Echinococcus* spp., and *T. solium* genomes. The seed sequences of each miR are in black and underlined.

Figure 3 shows the sequences and number of reads of the most abundant miRs in *T. solium* larvae; namely Tso-miR-10-5p (670475 reads), Tso-let-7-5p (184435 reads), Tso-miR-71-5p (107153 reads), and Tso-miR-4989-3p (94010 reads), followed by Tso-miR-61-3p, Tso-miR-125-5p, Tso-miR-2c-3p, and Tso-bantam-3p with more than 20000 reads. It also shows the sequences and number of reads of the most abundant miRs in *T. crassiceps* larvae; they were Tcr-miR-10-5p (907685 reads), Tcr-miR-4989-3p (199774 reads), Tcr-let-7-5p (118312 reads), and Tcr-miR-71-5p (116357 reads), followed by Tcr-miR-125- 5p, Tcr-miR-2c-3p, and Tcr-bantam-3p. The seed sequence is shown in the same figure.

**Figure 4:**
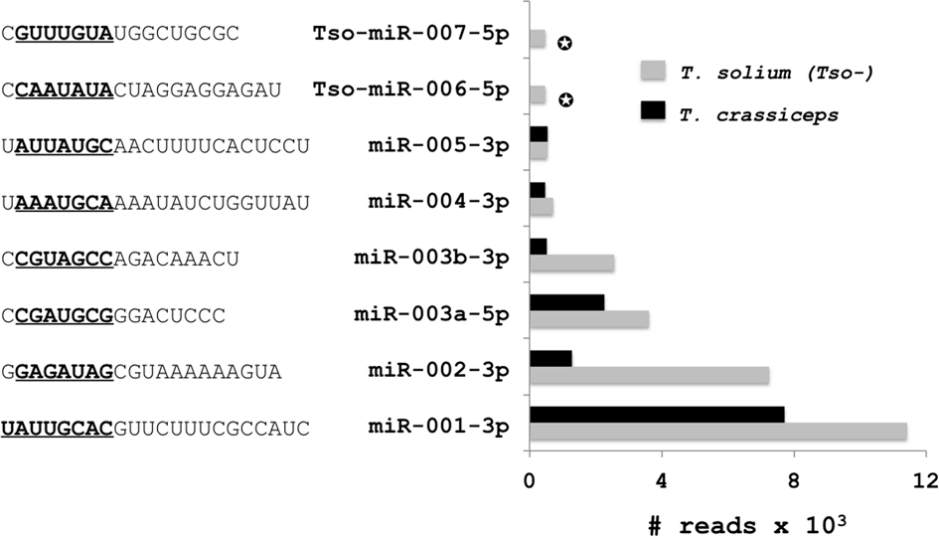
Sequences and reads of mature miRs within the hairpins unmapped to known helminths but mapped to the *T. solium* genome The cutoff for the number of copy sequences was 300 reads. The symbol ✪ shows miRs found only in the *T. solium* (Tso-) library. The seed sequences of each miR are in black and underlined.

The sequence, length, and identification of total known miRs found in both parasites are shown in Supplementary Table S1. We observed a similar abundance of miRs for both *Taenia* larvae, likewise, we also detected several miRs that were unknown in *T. solium* and *T. crassiceps* and that did not map to the genomes of other helminths different to genus *Taenia*; these miR sequences and copy numbers are shown in Supplementary Table S2, and the most abundant miRs in this group are shown in Figure 4. The most abundant genus-specific unknown miR (miR-001-3p) was identical in both *Taenia* larvae (11414 reads to *T. solium* and 7720 reads to *T. crassiceps*). Others abundant miRs were Tso-miR-002-3p (7252 reads) and Tcr-003-3p (2816 reads), where Tso-miR-001-3p represented more than 30% of the total of reads in this group. We also found some novel miRs in *T. solium* that were absent from *T. crassiceps*, but with a low frequency of ∼1000 reads (listed in Supplementary Table S3). Table 2 shows the location of the most abundant miRs and two genus-specific unknown miRs (miR-001-3p and miR-002-3p) on the *T. solium* genome. Notably, miR-71-5p and the miR-2 family were localized in the same cluster, as were miR-4989-3p and miR-277-3p in both species.

**Table 2:** Localization of miRs in the genome of *T. solium* (BioProject PRJNA170813), obtained by BLASTn homology search

miRNA	Length	Genome ID	Start/End
**miR-10-5p** **miR-10-3p**	22	pathogen_TSM_contig_00001	733529/733606
**let-7-5p**	22	pathogen_TSM_contig_01305	3774/3842
**miR-71-5p**	22	pathogen_TSM_contig_01703	213/2222
**miR-2c-3p**	21	pathogen_TSM_contig_01703	2240/2310
**miR-2b-5p**	24	pahogen_TSM_contig_01703	2346/2421
**miR-2b-3p**	23	pathogen_TSM_contig_00316	47891/47974
**miR-4989-3p** **miR-277-3p**	22	pathogen_TSM_contig_00002	355524/355602 355654/355741
**rniR-61-3p**	23	pathogen_TSM_contig_00547	54101/54189
**miR-125-5p**	22	pathogen_TSM_contig_00587	14115/14194
**bantam-3p**	22	pathogen_TSM_contig_00288	81838/81931
**miR-001-3p**	22	pathogen_TSM_contig_00022	149801/149908
**miR-002-3p**	20	pathogen_TSM_contig_03534, Pathogen_TSM_contig_03831	3328/34593365/3473310/415

Members of the same family are shown in the same row.

**Figure 5:**
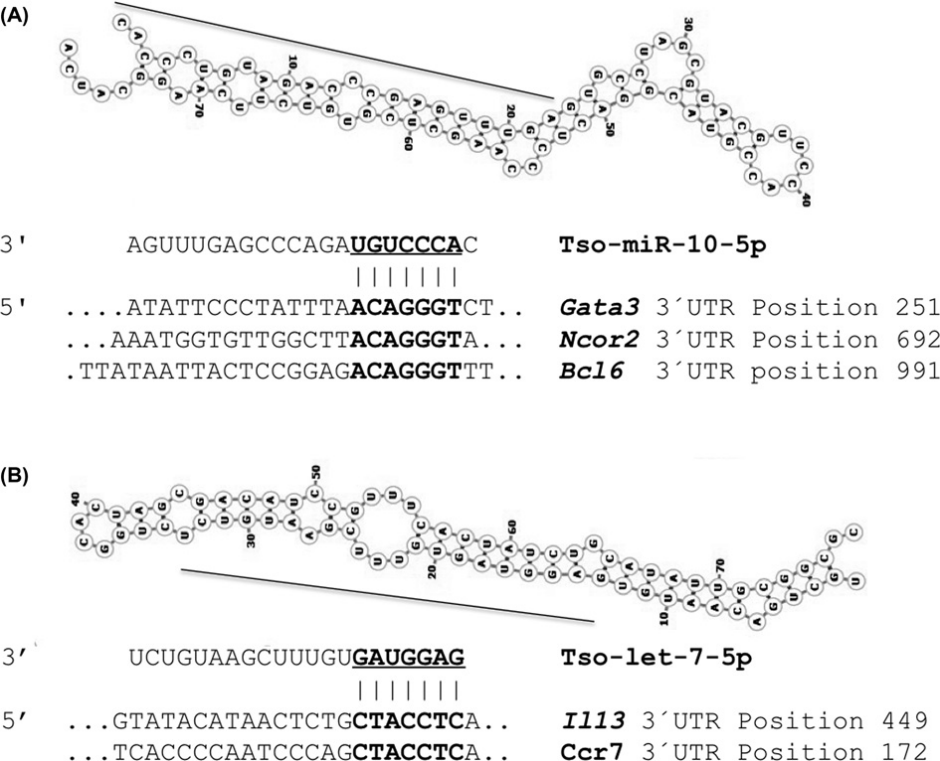
Secondary structures of the most abundant pre-miRs of ***T. solium*** and *T. crassiceps* and predicted immunological targets (**A**) miR-10-5p, and (**B**) let-7-5p. The black line on the hairpin structure shows the position of the miRs. The sequence alignments with the target transcripts show the sites of interaction of the miR seed sequence on the 3′-UTR.

Figure 5A shows the predicted hairpin structure for miR-10-5p with a minimum free energy of −26.50 kcal/mol, and its predicted immunologic targets, the transcriptional repressor B-cell lymphoma 6 (*Bcl6*), coding for a molecule involved in the stability or regulatory T cells [33], the nuclear receptor corepressor 2 (*Ncor2*), and the *trans*-acting T-cell-specific transcription factor (*Gata3*), which inhibit proliferation and promote apoptosis in diffuse large B-cell lymphomas [34]. Figure 5B shows the let-7-5p hairpin structure with a minimum free energy of −34.10 kcal/mol and the two predicted targets, the interleukin 13 (*Il13*) and de CC-chemokine receptor 7 (*Ccr7*) cytokines involved in anti-inflammatory response [35]. Similarly, Supplementary Figure S1 shows the predicted structure of novel pre-miR Tso-mir-001-3p, with the minimum free energy of −44.90 kcal/mol, and its predicted immunologic targets; namely, early T-cell activation antigen P60 (*Cd69*), an important regulator of the immune response highly expressed by memory and regulatory T cells in the gut, which is associated with negative regulation of Th1 and Th17-mediated immune response [36]. Notably, this gene is the principal target for this miR, matching at three positions in the 3′-UTR of its mRNA. Other targets of this miR were the TNF receptor-associated factor 3 (*Traf3*), which is involved in the signal transduction of CD40 and the regulation of NF-kB1 activation [37] and the suppressor of cytokine signaling 5 (*Socs5*), a cytokine-inducible negative regulator of cytokine signaling that plays a role as a specific negative regulator for Th2 differentiation [38]. Supplementary Figure S2 shows the secondary structure of Tso-mir-002-p3, with a free energy of −69.40 kca/mol, and the putative immunologic target, signaling lymphocytic activation molecule family member 1 (*Slamf1*) and the V-Set immunoregulatory receptor (*Vsir*). The *Slamf1* has a costimulatory effect on T cells and participates in the activation of B lymphocytes and some authors have suggested that may be involved in the generation of the adaptive and inflammatory immune responses [39], *Vsir* (VISTA), is a negative immune-checkpoint protein associated with suppression of T-cell response and suppression of cytokines like IL-2 [40]. Table 3 lists the other abundant *T. solium* and *T. crassiceps* miRs and their putative immunologic target genes, such as *Il10, Il12, Tnf, Mmd2, Tgfbr1, Irf4, Mapk4, Nfκb1*, and *Il23p40*, which are involved in immunologic regulatory functions in the innate and adaptive immune responses of the cells.

**Table 3:** Prediction of the putative immunologic gene targets of the most abundant *T. solium* and *T. crassiceps* miRs and their associated functions

miRNA	Target	miR-function associated
**milt-10-5p**	*Ncor2, Bc16, I112/I123p40*	Promote the Treg production, negative regulator of Th1 and Th17 T cell differentiation
**let-7-5p**	*I110, I113, Ccr7*	Promotes development of Th1, Th17 cells and IFN-γ -producing NKT cells
**bantam-3p**	*Mmd2, Tgfbr1*	Regulation of cell proliferation
**miR 125-5p**	*Tnf, Irf4*	Down-regulates pro-inflammatory signaling, promotes macrophage activation. Involved in WNT1 and TGF-β signaling, block the TNF biosynthesis
**m1R-9a-5p**	*Nfkb1, Mapk4*	Negative regulator of TLR4 signaling
**mix-001-3p**	*Cd69, Socs5, Traf3*	Putative involved in T-cell regulation
**mir-002-3p**	*Slamf1, Vsir*	Putative involved in regulation of T-cell cytokine production

### Uptake of miRs by peritoneal macrophages

**Figure 6:**
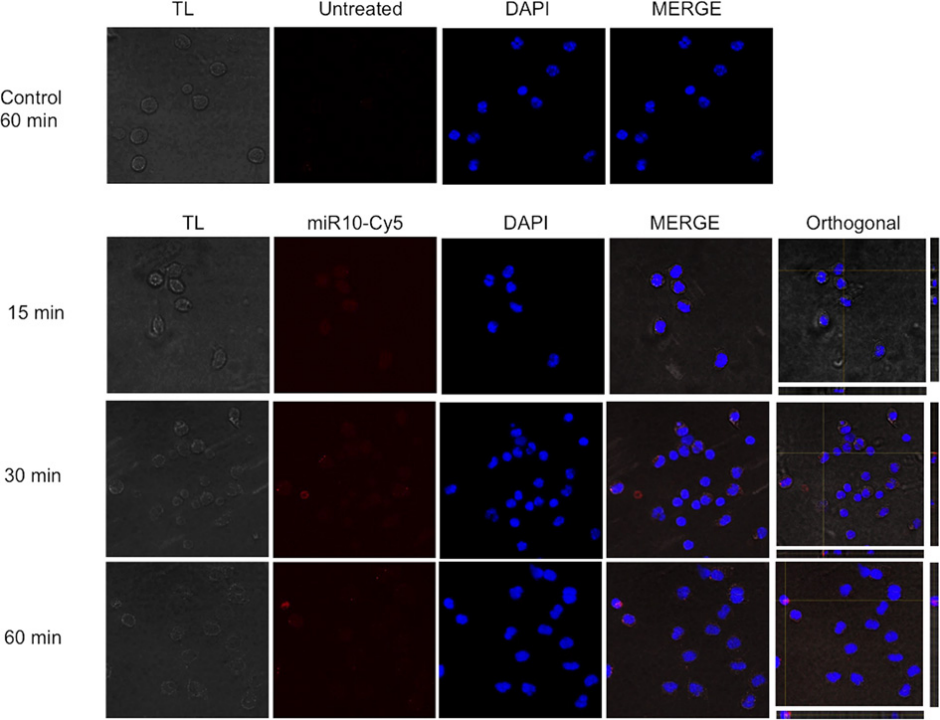
The miR uptake by murine peritoneal macrophages was observed by Leica FV 1000 confocal microscopy Cells were incubated at 15, 30, and 60 min without and with 100 nM of miR-10-5p coupled to Cy5 (miR10-Cy5, red). The DNA in the nucleus of the cells was stained with DAPI (blue). Transmitted light (TL) images of the macrophages were taken at each time point. The colocalization of all the components is shown in the merged images, and orthogonal images (single Z-plane) are shown for all the time points. Scale bars represent 20 μm.

For further assays, we chose miR-10-5p and let-7-p5 due to their abundance and predictive targets involved in the classical and alternative activation of macrophages, respectively. The M(N) macrophages were incubated with miR-10-5p-Cya5 (miR10-Cya5) or let-7-5p-Cya5 (let7-Cya5) *in vitro* and the internalization was monitored at different times by confocal microscopy. Figure 6 shows the presence of miR-10-Cya5 in the cytoplasm, where few faint red specks were observed at 15 min, while the images at 30 and 60 min, showed a clear pattern of red specks in the cytoplasm. A blue signal (DAPI) was observed in the nucleus of all macrophages. In addition, macrophages incubated without miRs showed no red signals. Orthogonal sections showed red specks inside the cytoplasm of cells. Similar results were observed with let7-Cya5 (Supplementary Figure S3). The quantification shows a significant increase in the fluorescence intensity inside the cells, >3 units at 30 and 60 min with respect to 15 min for miR10-Cya5, while the increase in let7-Cya5 had significance only between 15 and 60 min (Supplementary Figure S4).

### Effect of *T. solium* miRs on peritoneal macrophages

**Figure 7:**
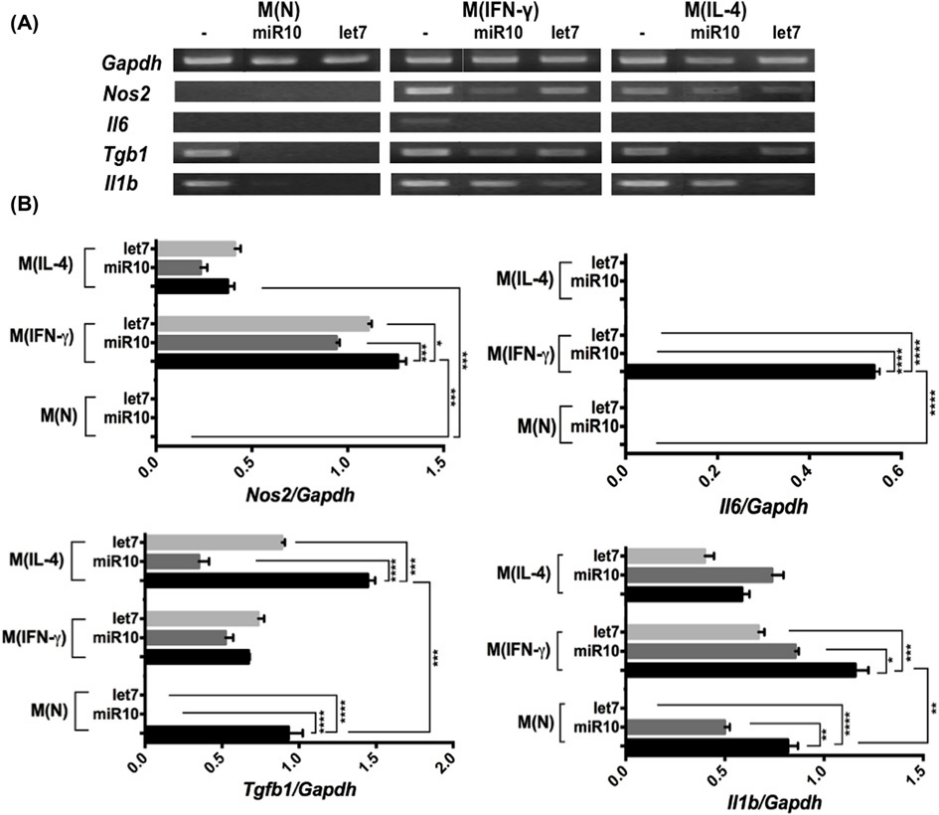
Effect of miR-10-5p (miR10) or let-7-5p (let7) on native or activated peritoneal macrophages (**A**) Representative Ethidium Bromide-stained gel showing the RT-PCR-amplified fragments from the *Gapdh, Nos2, Il6, Tgfb1*, and *Il1b* genes. For RT-PCR, total RNA was extracted from the culture of naive peritoneal macrophages M(N) and activated with IFNγ M(IFNγ) or with IL-4 M(IL-4), which were not treated (-) or treated with miR10 or let7. (**B**) Bars represent the mean ± SEM of the expression level of target genes normalized to that of *Gapdh*, analyzed with Kodak Digital Science 1D Image Analysis software. The data are representative of three independent experiments (*n*=3). *P<0.05; **P<0.01; ***P<0.001; ****P<0.0001, according to one-way ANOVA and Tukey’s multiple comparisons test.

**Figure 8:**
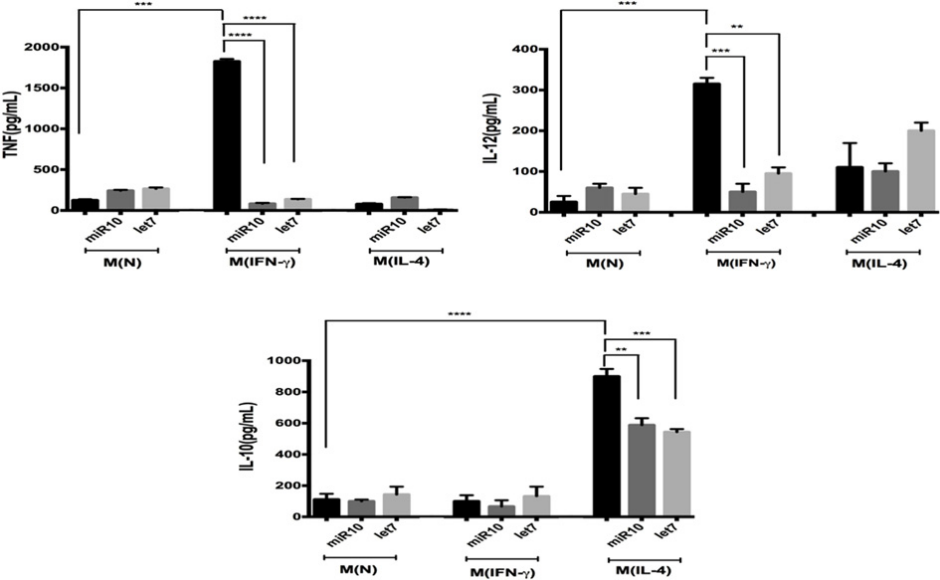
ELISA determination of cytokines (TNF, IL-12, and IL-10) secreted into the culture supernatant of macrophages: M(N), M(IFNγ), and M(IL-4) without treatment or treated with miR-10-5p (miR10) or let-7-5p (let7) (see Figure 7 for the definitions of the macrophage groups) The data are representative of three independent experiments (*n*=3). Bars represent the mean ± SEM. **P<0.01; ***P<0.001; ****P<0.0001, according to one-way ANOVA and Tukey’s multiple comparisons test.

The pattern of cytokines in M(N), M(IFN-γ), and M(IL-4) macrophages and the effect of miRs over them is shown in Figure 7. In M(N) macrophages only *Il1b* and *Tgfb1* were expressed, the treatment with IFN-γ induced an M1-like activation with a significant increase in *Nos2, Il6*, and *Il1b* expression, while the treatment with IL-4 increases the *Tgfb1* expression. Likewise, the treatment with the miRs altered the expression of this cytokines in all groups (Figure 7A,B). For instance, in the M(IFN-γ) macrophages the expression level of *Nos2* and *Il1b*, decreased significantly upon incubation with miR-10-5p or let-7-5p and the *Il6* expression, was blocked with the incubation of any of the miRs. In the M(N) macrophages, the *Il1b* expression decreased significantly upon incubation with miR-10-5p and was totally blocked with let-7-5p. In M(IL-4) macrophages. the *Il1b* expression increased moderately with miR-10 and decreased with let-7. Concerning *Tgfb1*, the expression was reduced by both miRs in M(N) and M(IL-4) macrophages but not was affected in the M(IFN-γ). The miR-10-5p showed a bigger effect over *Nos2* and *Tgfb1* and let-7-5p over *Il1b*. A constant expression of the *Gapdh* (housekeeping gene) was observed in all the three macrophage groups (Figure 7A). Likewise, in the culture supernatant (Figure 8), we observed a basal secretion of TNF, IL-12, and IL-10 in the M(N) macrophages, that is not significantly modified upon incubation with any of the miRs. As expected, the M(IFN-γ) macrophages increased the secretion of TNF and Il-12 significantly, but it is reduced at basal level upon incubation with both miRs. The M(IL-4) macrophages produced basal levels of this cytokines, and we did not find any statistical differences between treatments. In contrast, M(IL-4) macrophages increased strongly the secretion of IL-10, which is decreased by one-third upon incubation with any of the miRs.

## Discussion

In the present study, we described, as the first step, the miRNomes of the larvae of the closely related organisms *T. solium* and *T. crassiceps*. A total of 18.26 million reads of high quality for *T. solium* and 15.24 million for *T. crassiceps* were obtained, with a total of 335 miRs and 192 miRs, respectively. We found at least 40 miRs that were classified as known miRs, resembling the miRs of cestodes like *Echinococcus* genus with high identity (100% identity in more than 50% of miRs) and more than 100 unknown miRs (not found in other cestodes); similar sequences were shared by both the species, and we also found great similarities in the kinds, sizes, and distributions by copy number among the larvae of these two *Taenia* species. In addition, for both larvae, the most abundant miR was miR-10-5p, while the other abundant miRs were let-7-5p, miR-71-5p, and miR-4989-3p. It was in concordance with the results of other cestodes, such as *Mesocestoides corti, Echinococcus multilocularis*, and *Echinococcus canadensis* [41,42]. In contrast, in *Taenia* adult stage, miR-7-5p, miR-71, miR-277, miR-219-5p, and miR-2b-3p were predominant in *T. solium* and *T. asiatica*, and miR-71 and miR-219-5p were the most abundant in *T. saginata* [25,26]. Notably, miR-71 was localized in the same cluster with miR-2b and miR-2c, while miR-4849 was in the same cluster with miR-277; these clusters are conserved across Cestodes [24,43]. In addition, miR-71 is highly expressed in larvae and adults, and it has been involved in processes, such as longevity and neuron development, suggesting that it could be involved in important biological functions in the life cycle of *Taenia* genus [44,45]. Moreover, we found some putative miRs, present only in *T. solium* and others in *T. crassiceps*, but they all matched with the *T. solium* genome. This result could not be confirmed to *T. crassiceps* since the number of reads was low, and the *T. crassiceps* genome is not currently available. In our study, the predicted hairpin structures, the presence of homologs to Drosha, Dicer, and Pasha (as identified in the *T. solium* Genome Project) [46], and the differences in miR profiles between the *Taenia* larvae and adults suggest that: (1) the process for the synthesis of miRs is similar to that described for mammals, and (2) the expression of miRs is in accordance with the environment confronted by the larvae or adult in the hosts.

In our target prediction analysis, we found that *Taenia* larvae miR-10-5p, miR-125-5p, and Tso-miR-001 have target transcription factors and adaptor proteins that are involved in macrophage IRF/STAT pathways, such as *Cd69* and *Tnf*, involved in the IFN-signaling pathway, which regulate the expression of cytokine receptors, cell activation markers (CD38, CD69, CD97), and cell adhesion molecules that activate macrophages to secrete TNF to stimulate more macrophages through the TNFR1, leaving to classical activation [19,47,48]. Moreover, the targets of miR-125 and miR-9, such as *Irf4* and *Nfkb*, also are important in the classical activation of cells because TNF is mediated by canonical NF-κB and MAPK signaling that activates *Il1* and *Il6*, while IRF4-deficiency allows the generation of Foxp3^+^ Tregs cells [49]. In cysticercosis, Tregs induction seems to participate in the control of the inflammatory responses since a negative correlation between the percentage of peripheral Tregs and activated CD8^+^ and CD4^+^ T cells, along with a depressed T-cell proliferative response has been observed [50]. Likewise, the miR-10-5p targets, such as *Il12* and *Il23*, could interfere with the IL-12 family signaling pathway. In addition, let-7-5p showed a predicted target that encodes cytokines involved in M2 polarization, such as *Il10* that triggers M2c subtype phenotype and *Il13* that elicits the M2a phenotype [51]. In contrast, Tso-mir-002-3p has targets that participate in the activation of T and B lymphocytes, and therefore, is involved in the adaptive immune responses [39]. These analyses suggest an important role for *Taenia* miRs in the polarization of macrophages. Macrophages in cysticercosis promote a transient Th1 protective response with classical activated macrophages that is changed by parasite products to a Th2 permissive response with alternatively activated macrophages [14]. Moreover, studies in human and murine macrophages showed that miR-9, miR-127, miR-155, and miR-125b are associated with classical activation directly affecting *IFN*γ or *Bcl6* [19,20,52]. In addition, other miRs, such as miR-223, miR-34a, miR-132, miR-146a, miR-125a-5p, let-7c, and miR-124 promote alternative activation by regulating TLR4 and IL-4 signaling [21,22,52].

Already knowing that the more abundant *Taenia* miRs (miR-10-5p, let-7-5p) putatively have target genes of immune response and that macrophages in murine cysticercosis promote Th1 or Th2 responses [9,10]. We demonstrated by confocal microscopy that synthetic miR-10-5p and let-7-5p were internalized into the cytoplasm of M(N), M(IFN-γ), and M(IL-4) murine peritoneal macrophages *in vitro*. Notably, the incubation of M(IFN-γ) macrophages with miR-10-5p or let-7-5p significantly down-regulated the expression of *Il6, Il1b*, and TNF, IL-12 secretion (pro-inflammatory cytokines). Moreover, in the M(IL-4) macrophages, these miRs reduced the expression of cytokines involved in M2/Th2 differentiation. Even in our predicted analysis, let-7-5p targets IL-10 and moderately down-regulates this cytokine. However, our results for let-7-5p are in agreement with those observed for let7, let-7a, and let-7d that down-regulate IL-6 and IL-10 directly or TNF, IL-6, and IL-1β indirectly in MCF10A, fibroblasts, and breast cancer cells [53,54,55]. These results were important, because murine resistant to cysticercosis display high levels of TNF, IL-12, IL1-β, and NO during early infection (Th1 response), which is associated with the elimination of larvae [12]. On the other hand, high levels of pro-inflammatory cytokines (IL-6 and TNF) cause damage to the microglia promoting autoimmune and neurodegenerative diseases [56,57]. This tissue damage is also observed in human NCC at the beginning of larvae degeneration [2,3] and in pig NCC when they are treated with praziquantel [6]. In contrast, viable larvae are associated with a long initial asymptomatic phase that correlate with a undetectable inflammation in the Central Nervous System, presumably due to *T. solium* larvae factors prevent inflammation [58]. On the other hand, evidence exists that an sRNA-peptide of *T. solium* significantly reduces the inflammation around the larvae and decreases the antibody responses in the murine cysticercosis [59]. Therefore, the property of *Taenia* miRs, i.e. the down-regulation of pro-inflammatory cytokines in M(IFN-γ) macrophages could be used in the future to control tissue damage and the process of chronic neurodegeneration in Alzheimer, Parkinson, amyotrophic lateral sclerosis, multiple sclerosis, and NCC [60,61]. However, more studies are needed to be carried out to explore it further. The understanding the role of miRs in host–parasite relationship could help find other targets to develop new molecules for the regulation of inflammation [59]. Additionally, these miRs could be targets of miRNA inhibitors with the aim of favoring a protective response against the parasite. On the other hand, the abundant and specific *Taenia* miRs could be used as markers in the diagnosis of this parasitic disease [62].

Future studies are currently under way to evaluate the precise mechanism of the uptake of nude *Taenia* larvae miRs by macrophages and the time required for macrophage differentiation and also to determine the effect of *Taenia* unknown and specific miRs on immune cells.

## Conclusion

Our results show that larval miRNomes of *T. crassiceps* and *T. solium* are similar in composition. Both miR-10-p5 and let-7-5p, the most abundant miRs, are conserved in the larvae belonging to the *Taenia* genus, and strongly down-regulate the production of pro-inflammatory cytokines in macrophages M(IFN-γ) and moderately anti-inflammatory cytokines in macrophages M(IL-4). It suggests a new immunosuppressive mechanism that helps the larvae in their establishment and permanence inside the host. Furthermore, we propose that therapies based in the *Taenia* miRs could be employed in the control of larvae of cestode and inflammatory diseases.

## Supplementary Material

Supplementary Figures S1-S4 and Tables S1-S3

## Abbreviations

Bcl6: B-cell lymphoma 6
CNS: Central Nervous System
Cy5: Cyanine 5
DAPI: 4′,6-diamidino-2-phenylindole
Gapdh: glyceraldehyde 3-phosphate dehydrogenase
GATA3: GATA binding protein 3
IFN: interferon
Il1b: interleukin 1β
Il6: interleukin-6
IL-10: interleukin-10
IRF: interferon regulatory factor
miR: microRNA
NCC: neurocysticercosis
Ncor2: nuclear receptor corepressor 2
Nos2: nitric oxide synthase, inducible
PBS: phosphate-buffered saline
rRNA: ribosomal RNA
siRNA: small interfering RNA
Slamf1: signaling lymphocytic activation molecule family member 1
snoRNA: small nucleolar RNA
Socs5: suppressor of cytokine signaling 5
sRNA: small RNA
TLR: toll-like receptor
TNF: tumor necrosis factor
TREG: regulatory T cells
tRNA: transfer RNA
Vsir: V-Set immunoregulatory receptor
3′-UTR: 3′ untranslated region
