# Correction: *Taenia solium* and *Taenia crassiceps*: miRNomes of the larvae and effects of miR-10-5p and let-7-5p on murine peritoneal macrophages

**DOI:** 10.1042/BSR-2019-0152_COR

**Published:** 2021-06-28

The authors of the original article “*Taenia solium* and *Taenia crassiceps*: miRNomes of the larvae and effects of miR-10-5p and let-7-5p on murine peritoneal macrophages” (*Biosci Rep* (2019) **39**(11) BSR20190152), have realised that a splice had been introduced in Figure 7A of their article during their figure preparation. The original files used to create this figure have been assessed and approved by the Deputy Editor-in-Chief and the Editorial Board, and a version without this splice is presented below. The authors would also like to correct some typos in Table 3 of their article. The correct version of their table is also present in this Correction.

**Figure 7:**
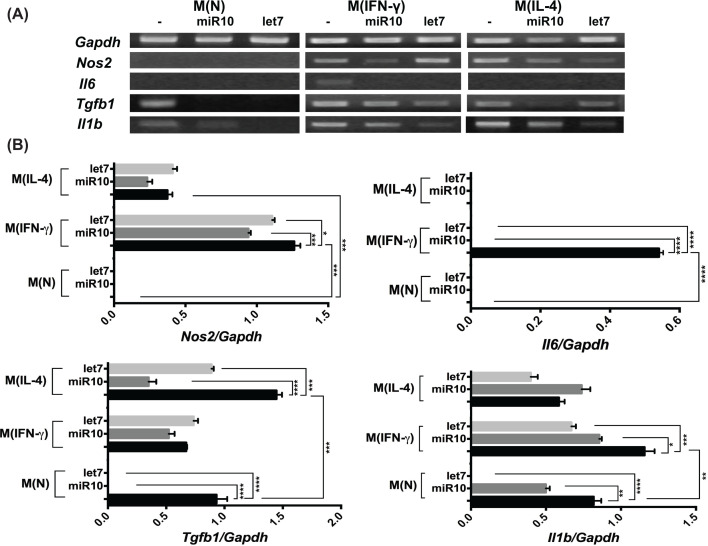
Effect of miR-10-5p (miR10) or let-7-5p (let7) on native or activated peritoneal macrophages (**A**) Representative Ethidium Bromide-stained gel showing the RT-PCR-amplified fragments from the *Gapdh, Nos2, Il6, Tgfb1*, and *Il1b* genes. For RT-PCR, total RNA was extracted from the culture of naive peritoneal macrophages M(N) and activated with IFNγ M(IFNγ) or with IL-4 M(IL-4), which were not treated (-) or treated with miR10 or let7. (**B**) Bars represent the mean ± SEM of the expression level of target genes normalized to that of *Gapdh*, analyzed with Kodak Digital Science 1D Image Analysis software. The data are representative of three independent experiments (*n* = 3). *P<0.05; **P<0.01; ***P<0.001; ****P<0.0001, according to one-way ANOVA and Tukey's multiple comparisons test.

**Table 3:** Prediction of the putative immunologic gene targets of the most abundant *T. solium* and *T. crassiceps* miRs and their associated functions

miRNA	Target	miR-function associated
**miR-10-5p**	*Ncor2, Bcl6, Il12/Il23p40*	Promote the T*reg* production, negative regulator of Th1 and Th17 T cell differentiation.
**let-7-5p**	*Il10, Il13, Ccr7*	Promotes development of Th1, Th17 cells and IFN-γ ‐producing NKT cells.
**bantam-3p**	*Mmd2, Tgfbr1*	Regulation of cell proliferation.
**miR-125-5p**	*Tnf, Irf4*	Down- regulates pro-inflammatory signaling, promotes macrophage activation. Involved in WNT1 and TGF-β signaling, block the TNF biosynthesis
**miR-9a-5p**	*Nfkb1, Mapk4*	Negative regulator of TLR4 signaling
**mir-001-3p**	*Cd69, Socs5, Traf3*	Putative involved in T cell regulation
**mir-002-3p**	*Slamf1, Vsir*	Putative involved in regulation of T cell cytokine production

The authors declare that these modifications do not affect the conclusions of their study.

