# Supplementary material for: Taenia solium and Taenia crassiceps: miRNomes of the larvae and effects of miR-10-5p and let-7-5p on murine peritoneal macrophages
Source: Biosci Rep. 2019 Nov 19;39(11):BSR20190152. doi: 10.1042/BSR20190152 (PMC6863767; doi:10.1042/BSR20190152)
Supplement: Supplementary Figures S1-S4 and Tables S1-S3 [file BSR-2019-0152_supp.pdf]

Fig. S1.

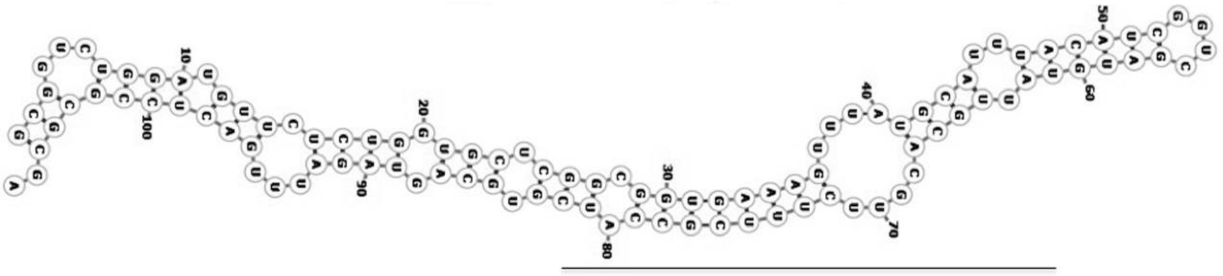

3´ CUACCGCUUUCUUG**CACGUUAU** **Tso-miR-001-3p**

||||||||

....AATAGTGTTGGAAAAT**GTGCAATA**TG *Cd69* 3´UTR position 758

...TAAATCAATTCTATTAC**GTGCAATA**CT *Cd69* 3´UTR position 870

...ATTTAAAATATTTTTAT**GTGCAATA**AA *Cd69* 3´UTR position 907

5´ ...CTTTCTAAAATTCAAGA**GTGCAAT**TTT *Traf3* 3´UTR position 461

..CCTACTTTAAAGCATTAT**GTGCAAT**TAA *Socs5* 3´UTR position 2070

Supplementary Fig. S1. Predicted structure for the pre-miR corresponding to Tso-mir- 001-3p. The mature miR sequence is underlined in the hairpin structure. The sequence alignments show the interaction between Tso-mir-001-3p and the 3′-UTR of the mRNA of Traf6, Socs5, and Cd69. Notably, the miR of Cd69 has two other sites for interaction. The putative seed and target interaction are shown in black.

Fig. S2.

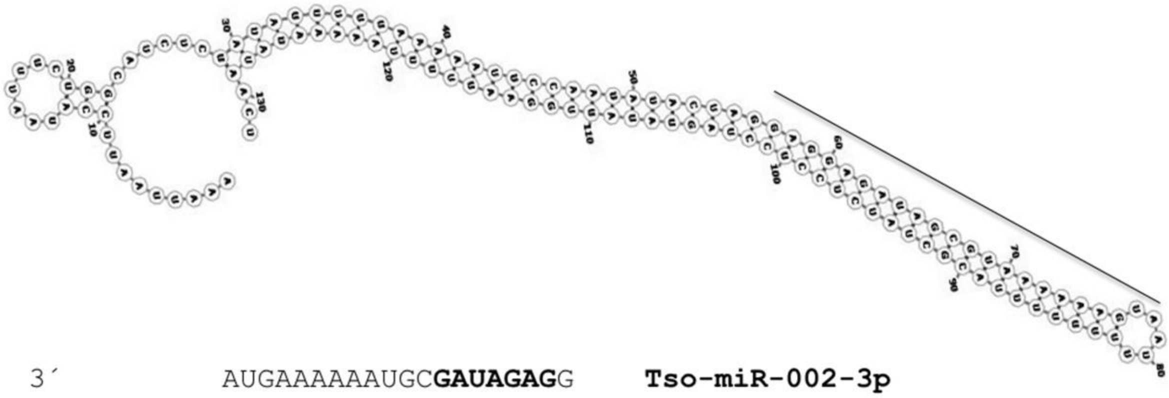

3´ AUGAAAAAAUGC**GAUAGAG**G **Tso-miR-002-3p**

|||||||

5´...ATGTGTATCACCCACTTC**CTATCTC**ACT ***Slamf1*** 3´UTR position 889

...AGATGCACAGACCCTCCC**CTATCTC**TTT **Vsir** 3´UTR position 156

...TTGGAGCGGGTGCTTTAT**CTATCTC**TGG **Vsir** 3´UTR position 2354

...**TCTC**TGGAGGGTCTCTAC**CTATCTC**CGA **Vsir** 3´UTR position 2375

Supplementary Fig. S2. Predicted structure for the pre-miR corresponding to Tso-mir- 002-3p. The mature miR sequence is underlined in the hairpin structure. The miR interacts with the 3′-UTR of the signaling lymphocytic activation molecule family member 1 (Slamf1) and V-Set immunoregulatory receptor (Vsir). The putative seed and target interaction are shown in black.

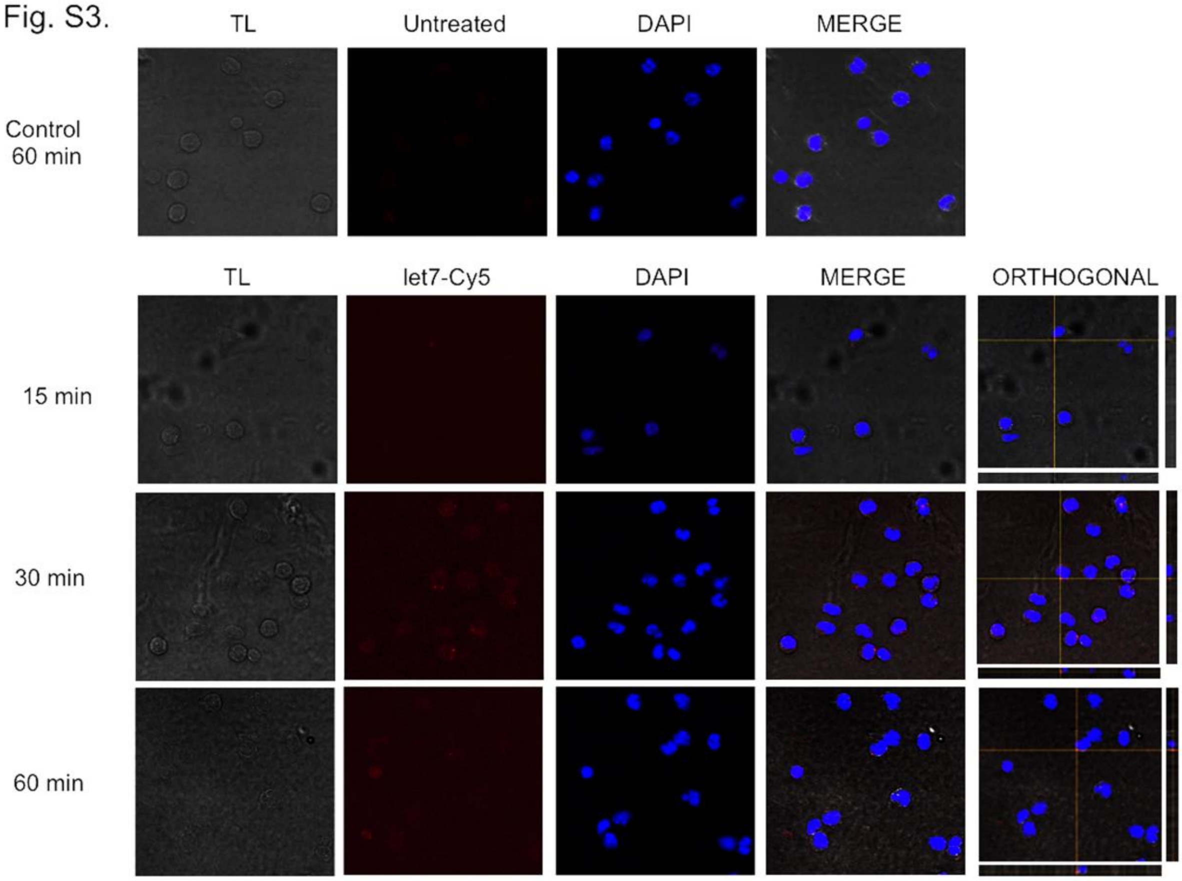

Supplementary Fig. S3. The miR uptake by murine peritoneal macrophages was observed by Leica FV 1000 confocal microscopy. Cells were incubated at 15, 30, and 60 min without and with 100 nM of let-7-5p coupled to Cyanine 5 (let7-Cy5, red). The DNA in the nucleus of the cells was stained with DAPI (blue). Transmitted light (TL) images of the macrophages were taken at each time point. The colocalization of all the components is shown in the merged images, and orthogonal images (single Z-plane) are shown for all the time points. Scale bars represent 20 µm.

Fig. S4

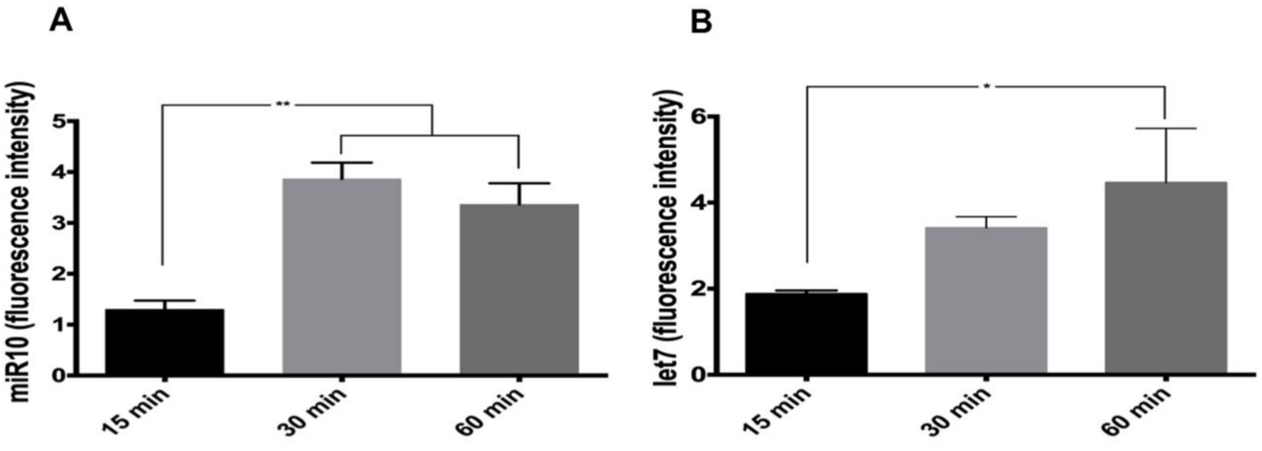

Supplementary Fig. S4. Fluorescence quantification. Bars represent the mean ± SEM of intensity of fluorescence. Data were obtained from cells incubated 15, 30, and 60 min with 100 nM of: (A) miR-10-5p coupled to Cyanine 5 (miR10) or (B) let-7-5p coupled to Cyanine 5 (let7), as shown in the Fig. 6 and Fig. S3

Supplementary Table S1. List of known miRs found in Taenia solium and Taenia crassiceps larvae. The homologous miRs for each species are in the same row. The miRs not detected in one species are shown as empty spaces. The sequence, length, and the number of copies are shown. The characterized miRs are shown in black.

Supplementary Table S2. List of unknown miRs found in Taenia solium and Taenia crassiceps larvae. The homologous miRs for each species are in the same row. The sequence, length, and the number of copies are shown. The characterized miRs are shown in black.

Supplementary Table S3. List of unknown miRs found in Taenia solium and Taenia crassiceps larvae that do not have homology with miRs of other species. The sequence, length, and the number of copies are shown.

Supplementary Table S1.

Taenia solium				*Taenia crassiceps*			
miR_name	miR_seq	lenght	#copy of the miRNA	miR_name	miR_seq	lenght	#copy of the miRNA
**Tso-let-7-5p**	**TGAGGTAGTGTTTCGAATGTCT**	**22**	**184435**	Tcr-let-7-5p	TGAGGTAGTGTTTCGAATGTCT	22	118312
**Tso-miR-10-5p**	**CACCCTGTAGACCCGAGTTTGA**	**22**	**670475**	Tcr-miR-10-5p	CACCCTGTAGACCCGAGTTTGA	22	907685
Tso-miR-71-5p_R+1	TGAAAGACGATGGTAGTGAGAT	22	107153	Tcr-miR-71-5p_R+1	TGAAAGACGATGGTAGTGAGAT	22	116357
Tso-miR-4989-3p	AAAATGCACCAACTATCTGAGA	22	94010	Tcr-miR-4989-3p	AAAATGCACCAACTATCTGAGA	22	199774
tso-miR-61-3p_R+1_2ss12TA21TA	TGACTAGAAAGAGCACTCACATC	23	51433	Tcr-miR-61-3p_R+1_2ss12TA21TA	TGACTAGAAAGAGCACTCACATC	23	26423
Tso-miR-125-5p	TCCCTGAGACCCTAGAGTTGTC	22	37016	Tcr-miR-125-5p	TCCCTGAGACCCTAGAGTTGTC	22	19171
Tso-miR-277-3p	TAAATGCATTTTCTGGCCCGTA	22	27402	Tcr-miR-277-3p	TAAATGCATTTTCTGGCCCGTA	22	22241
Tso-miR-2c-3p_R+1	TCACAGCCAATATTGATGAAC	21	24693	Tcr-miR-2c-3p_R+1	TCACAGCCAATATTGATGAAC	21	19228
Tso-bantam-3p_2ss15AC21GA	TGAGATCGCGATTACAGCTGAT	22	22834	Tcr-bantam-3p_2ss15AC21GA	TGAGATCGCGATTACAGCTGAT	22	52405
Tso-miR-1b-3p_R-1_1ss21GA	TGGAATGTTGTGAAGTATGTA	21	19732	Tcr-miR-1b-3p_R-1_1ss21GA	TGGAATGTTGTGAAGTATGTA	21	6258
Tso-mir-4988-p3	GGGACGGAAGTCTGAAAGGTTT	22	18260	Tcr-mir-4988-p3	GGGACGGAAGTCTGAAAGGTTT	22	8351
Tso-miR-745	TGCTGCCTGGTAAGAGCTGTGA	22	17190	Tcr-miR-745	TGCTGCCTGGTAAGAGCTGTGA	22	14306
Tso-miR-87-3p	GTGAGCAAAGTTTCAGGTGTGC	22	14740	Tcr-miR-87-3p	GTGAGCAAAGTTTCAGGTGTGC	22	7468
Tso-miR-1	TGGAATGTTGTGAAGTATGT	20	12918	Tcr-miR-1	TGGAATGTTGTGAAGTATGT	20	6405
Tso-miR-2162-3p_2ss12GT21TC	TATTATGCAACTTTTCACTCC	21	10467	Tcr-miR-2162-3p_2ss12GT21TC	TATTATGCAACTTTTCACTCC	21	4230
Tso-miR-2a_R+1	AATCACAGCCCTGCTTGGAACCA	23	10088	Tcr-miR-2a_R+1	AATCACAGCCCTGCTTGGAACCA	23	16965
Tso-miR-190_R+1	AGATATGTTTGGGTTACTTGGTGA	24	6240	Tcr-miR-190	AGATATGTTTGGGTTACTTGGTG	23	18153
Tso-miR-4989_R+1	AAAATGCACCAACTATCTGAGAA	23	5428	Tcr-miR-4989_R+1	AAAATGCACCAACTATCTGAGAA	23	25446
Tso-miR-2b_R+2	TATCACAGCCCTGCTTGGGACACT	24	4140	Tcr-miR-2b_R+2	TATCACAGCCCTGCTTGGGACACA	24	2519
Tso-mir-9-p3	CAAGGCTAGATTTCCAAACAAA	22	2696	Tcr-mir-9-p3_1ss4GA	CAAAGCTAGATTTCCAAACAAA	22	1847
Tso-miR-281-3p_R-2	TGTCATGGAGTTGCTCTCTA	20	2626	Tcr-miR-281-3p_R-2	TGTCATGGAGTTGCTCTCTA	20	3291
Tso-miR-9_R+1	TCTTTGGTTATCTAGCTGTGTGA	23	2418	Tcr-miR-9_R+1	TCTTTGGTTATCTAGCTGTGTGT	23	7826
tso-let-7-p3	ACATCCGTTTCACTATCTGCATA	23	1923	Tcr-let-7-p3	ACATCCGTTTCACTATCTGCATA	23	1665
Tso-miR-277_R+5	TAAATGCATTTTCTGGCCCGTAATT	25	1738	Tcr-miR-277_R+5	TAAATGCATTTTCTGGCCCGTAATT	25	3487
Tso-miR-219_R-1	TGATTGTCCATTCGCATTTCTT	22	1722	Tcr-miR-219_R-1	TGATTGTCCATTCGCATTTCTT	22	467
Tso-mir-2b-p5_1ss7AG	TGTCCCGAGGGTGGTGATCTACT	23	1288	Tcr-mir-2b-p5_1ss7AG	TGTCCCGAGGGTGGTGATCTACT	23	3006
Tso-miR-87-3p	GTGAGCAAAGTTTCAGGTGT	20	408	Tcr-miR-87-3p	GTGAGCAAAGTTTCAGGTGT	20	105
Tso-miR-7	TGGAAGACTGGTGATATGTTGT	22	334	Tcr-miR-7	TGGAAGACTGGTGATATGTTGT	22	672
Tso-miR-219-5p_R+2	TGATTGTCCATTCGCATTTCTTGT	24	229	Tcr-miR-219-5p_R+3	TGATTGTCCATTCGCATTTCTTGTT	25	383
tso-mir-125-p3	CAACTCTAATGTCCCGGGTTA	21	219	Tcr-mir-125-p3	CAACTCTAATGTCCCGGGTTA	21	155
Tso-mir-71-p3	TCTTGCTACTCCGTCTTTTCCTA	23	189	Tcr-mir-71-p3	TCTTGCTACTCCGTCTTTTCCTA	23	94
Tso-miR-2c-5p_R-2	TCGTCAACATTGCCTGTAGA	20	170	Tcr-miR-2c-5p_R-2	TCGTCAACATTGCCTGTAGA	20	189
Tso-mir-10-p3	AAGCTCGTGTCTTCAAGGAATCA	23	162	Tcr-mir-10-p3	AAGCTCGTGTCTTCAAGGAATC	22	294
Tso-miR-71a-5p_R+2	TGAAAGACGATGGTAGTGAGATG	23	142	Tcr-miR-71a-5p_R+2	TGAAAGACGATGGTAGTGAGATG	23	500
Tso-miR-96_R+2	ATTGGCACTTTTGGAATTGTCAC	23	128	Tcr-miR-96_R+2	ATTGGCACTTTTGGAATTGTCAC	23	160
Tso-miR-133a-3p_1ss10GT	TTGGTCCCCTTCAACCAGCTGT	22	93	Tcr-miR-133a-3p_L+1_1ss11GT	TTTGGTCCCCTTCAACCAGCTGT	23	291
Tso-miR-124b-3p	TAAGGCACGCGGTGAATACC	20	75	Tcr-miR-124b-3p	TAAGGCACGCGGTGAATACC	20	158
Tso-mir-307-p3	TCACAACCTACTTGATTGAGGGG	23	74	Tcr-miR-307_R+3	TCACAACCTACTTGATTGAGGGG	23	70
Tso-miR-87-5p_1ss3AG	CCGCCTGTCATTTTGCTCGAACC	23	64	Tcr-miR-87-5p_1ss3AG	CCGCCTGTCATTTTGCTCGAACC	23	205
Tso-mir-190-p3	CCAGTGACCAAACATATTCAC	21	55	Tcr-mir-190-p3	CCAGTGACCAAACATATTC	19	47
Tso-miR-124b-5p_R-1_1ss8AG	GTATTCTGCGCGATGTCTTGGT	22	54	Tcr-miR-124b-5p_2ss8AG23AT	GTATTCTGCGCGATGTCTTGGTT	23	9
Tso-mir-277a-p3_2ss11GA20AT	TAAATGCAAAATATCTGGTT	20	51	Tcr-mir-277a-p3_2ss11GA20AT	TAAATGCAAAATATCTGGTT	20	35
Tso-miR-749_R+8_1ss11GT	GCTGGGATGATCCTCGGTGGTCCGGGGTG	29	50	Tcr-miR-749_R+6_1ss11GT	GCTGGGATGATCCTCGGTGGTCCGGGG	27	48
Tso-miR-10-5p_L-1	ACCCTGTAGACCCGAGTTTGG	21	48	Tcr-miR-10-5p_R+1_1ss1AC	CACCCTGTAGACCCGAGTTTGGA	23	193
Tso-miR-2b-3p_R-5_1ss19GA	TATCACAGCCCTGCTTGGA	19	47	Tcr-mir-2b-p3_2ss18GA20AT	TATCACAGCCCTGCTTGAAT	20	1
Tso-miR-4988_R+3_1ss15AG	ACCTATCACACTTCGGTCCAGT	22	44	Tcr-miR-4988_R+3	ACCTATCACACTTCAGTCCAGT	22	18
Tso-mir-4989-p5	TGGGTAGTCGTTGCATTTCCA	21	42	Tcr-mir-4989-p5	TGGGTAGTCGTTGCATTTCC	20	11
Tso-mir-153-p5_1ss14GT	ATGCTTACGAGACTTGCACTC	21	27	Tcr-mir-153-p5_1ss14GT	ATGCTTACGAGACTTGCACTC	21	5
Tso-miR-125c-5p	TCCCTGAGACCTTAGAGTTGTC	22	27	Tcr-miR-125c-5p_R-1	TCCCTGAGACCTTAGAGTTGT	21	6
Tso-miR-10a-3p_1ss1AT	TAATTCGAGTCAACAGGGTCGT	22	19	Tcr-miR-10a-3p_1ss1AT	TAATTCGAGTCAACAGGGTCGT	22	5
Tso-miR-124a_R-1	TAAGGCACGCGGTGAATGCC	20	17	Tcr-miR-124a_R-1	TAAGGCACGCGGTGAATGCC	20	22
Tso-miR-7-5p	TGGAAGACTGGTGATATGTTGTT	23	10	Tcr-miR-7-5p	TGGAAGACTGGTGATATGTTGTT	23	56
Tso-mir-2152-p3	ATTCTCAAACTTTAAAT	17	8	Tcr-mir-2152-p3	TCAAACTTTAAATGGG	16	5
Tso-miR-2f-3p_R-3	TCACAGCCAATATTGATA	18	8	Tcr-miR-2f-3p_R-3	TCACAGCCAATATTGATA	18	7
Tso-miR-2c-3p_R+1_1ss10AG	TCACAGCCAGTATTGATGAAC	21	7	Tcr-miR-2c-3p_R-4_1ss11GC	TATCACAGCCCTGCTTAA	18	1
Tso-mir-2a-p5_1ss8GA	CGTTCCAAGGGGATGTGAGTTTA	23	6	Tcr-mir-2a-p5_1ss8GA	CGTTCCAAGGGGATGTGAGTTT	22	6
Tso-miR-153	TTGCATAGTCTCATAAGTGCCA	22	6	Tcr-miR-153	TTGCATAGTCTCATAAGTGCCA	22	3
Tso-mir-1992-p3_2ss12TA20CA	TCAGCAGTTGTACCATTGAAA	21	5	Tcr-mir-1992-p3_2ss12TA20CA	TCAGCAGTTGTACCATTGAAA	21	4
Tso-mir-133b-p3_2ss12CT20AC	TTGGTCCCCATTAACCAGCC	20	5	Tcr-mir-133b-p3_2ss12CT20AC	TTGGTCCCCATTAACCAGCC	20	13
Tso-miR-281-3p_R+3	TGTCATGGAGTTGCTCTCTATAATA	25	4	Tcr-miR-281-3p_R+3	TGTCATGGAGTTGCTCTCTATAATA	25	4
Tso-mir-1b-p5_2ss2AG23TA	TGCTTCCCAATAGGCCATAGAGAA	24	4	Tcr-mir-1b-p5_2ss2AG25AT	TGCTTCCCAATAGGCCATAGAGTAT	25	1
Tso-mir-2161-p5_2ss8TG17CA	TGCTTGCGTAGGTTGAA	17	4	Tcr-mir-2161-p5_2ss8TG17CA	TGCTTGCGTAGGTTGAA	17	1
Tso-miR-36c-3p_R+1_1ss18AC	TCACCGGGTAGACATTCCTTG	21	4	Tcr-mir-36a-p3_1ss18AC	TCACCGGGTAGACATTCCTT	20	10
Tso-miR-2b-3p_R-3_2ss18GA21CA	TATCACAGCCCTGCTTGAAAA	21	3				
Tso-mir-87-p5_2ss22GA24GA	CCTGTCATTTTGCTCGAACCAAAA	24	2	Tcr-mir-87-p5_1ss21GA	GTCATTTTGCTCGAACCAGAATT	23	1
Tso-mir-219-p3	AGATGTGTGTATGGGCATCGCA	22	2				
Tso-mir-125-p3_1ss23GT	CAACTCTAATGTCCCGGGTTATTA	24	2	Tcr-mir-125-p3_2ss23GT24AT	CAACTCTAATGTCCCGGGTTATTT	24	2
Tso-mir-749-p3_2ss26AG28TC	TCCGGGGTGCAGGCTTCAAACCTGTGGC	28	2	Tcr-mir-749-p3	TCCGGGGTGCAGGCTTC	17	10
Tso-miR-184b-3p_2ss20GT22GC	GGGACGGAAGTCTGAAAGGTACT	23	2	Tcr-miR-184b-3p_1ss20GT	GGGACGGAAGTCTGAAAGGTAGT	23	1
Tso-miR-2a_R+5	AATCACAGCCCTGCTTGGAACCAAAAT	27	2	Tcr-miR-2a_R+3	AATCACAGCCCTGCTTGGAACCTTA	25	4
Tso-miR-7d-5p_R+1_1ss13AG	TGGAAGACTAGTGATTTTGTTGTT	24	2	Tcr-miR-7d-5p_1ss13AG	TGGAAGACTAGTGATTTTGTTGT	23	2
Tso-miR-7-5p_R+2_2ss10TA18AT	TGGAAGACTAGTGATTTTGTTGTT	24	2	Tcr-miR-7-5p_R+2_2ss10TA18AT	TGGAAGACTAGTGATTTTGTTGTT	24	7
Tso-mir-125-p3_2ss22TA23GA	CAACTCTAATGTCCCGGGTTAAAA	24	2	Tcr-mir-125-p3_2ss23GA25GT	CAACTCTAATGTCCCGGGTTATAAT	25	2
Tso-miR-2b-3p_R-1_1ss12TA	TCACAGCCAATATTGATGAGA	21	2	Tcr-miR-2b-3p_R+5	TATCACAGCCCTGCTTGGGACACAATCTA	29	2
Tso-mir-1-p5_2ss17GA18TA	CCCAATAGGCCATAGAAAAA	20	1				
Tso-mir-124a-p5_2ss24CA26GT	GCGTTCAACCTCGGAGCCTTTGTAAT	26	1	Tcr-mir-124a-p5	GCGTTCAACCTCGGAGCCTTTGT	23	1
Tso-mir-745-p5_1ss3AG	CCGGTTCTTATCGGGTATATCATG	24	1	Tcr-mir-745-p5_1ss3AG	CCGGTTCTTATCGGGTATATCATG	24	2
Tso-mir-277-p5_2ss15AG28TC	ATGCTGCCTCTATGGGATCCACTTATGCTTG	31	1	Tcr-mir-277-p5_1ss28TC	ATGCTGCCTCTATGAGATCCACTTATGCTTG	31	5
Tso-miR-7-5p_R-1_3ss17TG18AG19GA	TGGAAGACTTGTGATTGGATT	21	1	Tcr-miR-7-5p_2ss18AG19GA	TGGAAGACTTGTGATTGGATTGTT	24	1
Tso-mir-2c-p5_2ss20CA21AT	CGTCAACATTGCCTGTAGAATC	22	1				
Tso-miR-7-5p_R+3	TGGAAGACTGGTGATATGTTGTTCAT	26	1	Tcr-miR-7-5p_R+2	TGGAAGACTGGTGATATGTTGTTTT	25	2
Tso-miR-1b-3p_R+1	TGGAATGTTGTGAAGTATGTGCC	23	1	Tcr-miR-1b-3p_2ss1TC22CG	CGGAATGTTGTGAAGTATGTGG	22	1
Tso-miR-190a-5p_R+2_1ss16AC	AGATATGTTTGGTTACTTGGTGATA	25	1	Tcr-miR-190_R+5	AGATATGTTTGGGTTACTTGGTGATATG	28	1
Tso-miR-124c-3p_R+2_1ss20CA	TAAGGCACGCGGTGAATGCAAGA	23	1	Tcr-miR-124-3p_1ss20CA	TAAGGCACGCGGTGAATGTAA	21	1
Tso-miR-9a-5p_1ss10TA	TCTTTGGTTATCTAGCTGTATGAT	24	1	Tcr-miR-9_R+2_1ss22GT	TCTTTGGTTATCTAGCTGTGTTGG	24	1

Supplementary Table S1.

Tso-miR-2b-3p_R+4	TATCACAGCCCTGCTTGGGACACATCTT	28	1	Tcr-miR-2b-3p_R-1_1ss12TA	TCACAGCCAATATTGATGAGA	21	1
Tso-mir-4988-p3_2ss25AT26CA	GGGACGGAAGTCTGAAAGGTTTAATAA	27	1				
Tso-mir-10b-p5_2ss1AT17GA	TACCCTGTAGATCCGAATTT	20	1	Tcr-mir-10b-p5_1ss10TC	CCCTGTAGACCCGAGTTTGATTA	23	5
Tso-miR-2162-3p_R+2_1ss12GT	TATTATGCAACTTTTCACTCTAT	23	1				
Tso-miR-10-5p_2ss10GC22GA	AACCCTGTACACCCGAGTTTGA	22	1				
Tso-mir-2f-p3_2ss11AC17CA	TATCACAGCCCTGCTAAA	18	1				
Tso-miR-190-5p_L-1R+2_1ss9AT	GATATGTTTGGGTTACTTGGTGTC	24	1	Tcr-miR-190-5p_R+1_1ss9AT	TGATATGTTTGGGTTACTTGGTGT	24	2
Tso-miR-219-5p_L-2R+3	ATTGTCCATTCGCATTTCTTATA	23	1				
Tso-mir-277c-p3_2ss11AT17TA	TAAATGCATTTTCTGGAAT	19	1	Tcr-miR-277c-3p_R-4_1ss9TA	TAAATGCAATATCTGGTA	18	6
Tso-miR-190-5p_R-4_1ss1TA	AGATATGTTTGGGTTACAA	19	1	Tcr-miR-190-5p_R-4_1ss1TA	AGATATGTTTGGGTTACAA	19	2
Tso-miR-71_L+3	TGGTGAAAGACGATGGTAGTGAGA	24	1	Tcr-miR-71_L+1R+3	GTGAAAGACGATGGTAGTGAGATTA	25	2
Tso-miR-9_R+3	TCTTTGGTTATCTAGCTGTGTGTGA	25	1	Tcr-miR-9_R+2_1ss22GT	TCTTTGGTTATCTAGCTGTGTTGG	24	1
Tso-mir-2c-p3_2ss11GC19GT	TATCACAGCCCTGCTTAAT	19	1				
Tso-mir-281-p5_2ss12TG17TC	AATGAAAATCTGCGAACT	18	1				
Tso-mir-4989-p3_3ss18TG19GC20AT	AAAATGCACCAACTATCGCTGAT	23	1				
Tso-miR-10b-5p_2ss10GC17AG	AACCCTGTACACCCGAGTTTGA	22	1	Tcr-miR-10b-5p_R+1_2ss1AC22AT	CACCCTGTAGACCCGAATTTGTT	23	1
Tso-miR-71a_R+4	TGAAAGACGATGGTAGTGAGATGAC	25	1	Tcr-miR-71a_R+3_1ss21AT	TGAAAGACGATGGTAGTGAGTTGT	24	1
Tso-miR-87a-3p_L+1R+2_1ss16AG	GTGAGCAAAGTTTCAGGTGTAAA	23	1	Tcr-mir-87a-p3_3ss16AT21AG23AT	GTGAGCAAAGTTTCATGTGTGTTTA	25	1
Tso-miR-2b_R+6	TATCACAGCCCTGCTTGGGACACTTGAT	28	1	Tcr-miR-2b_R+6	TATCACAGCCCTGCTTGGGACACATCTT	28	1
Tso-miR-9_R+3	TCTTTGGTTATCTAGCTGTGTGTGA	25	1	Tcr-mir-9a-p5_3ss10TG20AG23AT	TCTTTGGTTGTCTAGCTGTGTGTTT	25	1
Tso-mir-281-p3_2ss16CA17TC	TGTCATGGAGTTGCTACT	18	1	Tcr-mir-281-p3_2ss17TC19CA	TGTCATGGAGTTGCTCCTA	19	2
Tso-mir-2c-p3_2ss2AT17CA	ATATGTCACAGCCAATATTGATG	23	1				
Tso-miR-125c-5p_R+3	TCCCTGAGACCTTAGAGTTGTCTTT	25	1	Tcr-miR-125c-5p_R+1_1ss22CG	TCCCTGAGACCTTAGAGTTGTGA	23	1
Tso-mir-2d-p3_2ss11GC19AT	TATCACAGCCCTGCTTTAT	19	1	Tcr-mir-2d-p3_2ss11GC19AT	TATCACAGCCCTGCTTTAT	19	2
Tso-miR-125_R+5	TCCCTGAGACCCTAGAGTTGTCGTTAT	27	1	Tcr-mir-125a-p5_2ss17AT18TC	TCCCTGAGACCCTTTGTC	18	1
Tso-mir-4988-p3_2ss25AT26CA	GGGACGGAAGTCTGAAAGGTTTAATAA	27	1	Tcr-mir-4988-p3_2ss23AG26CG	GGGACGGAAGTCTGAAAGGTTTGAAGA	27	1
Tso-miR-219-5p_L-2R+4	ATTGTCCATTCGCATTTCTTATA	23	1	Tcr-miR-219-5p_R+4	TGATTGTCCATTCGCATTTCTTGTAA	26	13
Tso-miR-2a-3p_R+4_1ss10GA	TCACAGCCAATATTGATGAACGGTAT	26	1	Tcr-miR-2a-3p_R+4_1ss10GA	TCACAGCCAATATTGATGAACGGTAT	26	1
Tso-miR-71a-5p_R+4	TGAAAGACGATGGTAGTGAGATGAC	25	1	Tcr-miR-71a-5p_R+3_1ss21AT	TGAAAGACGATGGTAGTGAGTTGT	24	1
Tso-mir-2c-p3_2ss11GC19GT	TATCACAGCCCTGCTTAAT	19	1	Tcr-miR-2c-3p_R-4_1ss11GC	TATCACAGCCCTGCTTAA	18	1
Tso-mir-8457-p5_1ss11TA	TCGGTGGTGTAGTGA	15	1	Tcr-mir-8457-p5_2ss13TA17AG	GCTCGGTGGTGTAGTGG	17	1
Tso-mir-8a-p3_2ss11AG17CA	TAATACTGTCGGGTAAAGAT	20	1				
Tso-miR-190-5p_L-1R+2_1ss9AT	GATATGTTTGGGTTACTTGGTGTC	24	1	Tcr-miR-190-5p_R+1_1ss9AT	TGATATGTTTGGGTTACTTGGTGT	24	2
Tso-miR-2b_R+6	TATCACAGCCCTGCTTGGGACACTTGAT	28	1	Tcr-miR-2b_R+6	TATCACAGCCCTGCTTGGGACACATCTT	28	1
Tso-miR-745-3p_R+1	TGCTGCCTGATAAGAGCTGTGG	22	1	Tcr-miR-745-3p_R+6_1ss10AG	TGCTGCCTGGTAAGAGCTGTGAAGAGG	27	1
Tso-miR-10a-5p_R-2_2ss1AC21GA	CACCCTGTAGATCCGAGTTAA	21	1	Tcr-mir-10a-p5_2ss1AT17GA	TACCCTGTAGATCCGAATT	19	3
				Tcr-miR-2a-3p_R+3_1ss10GA	TCACAGCCAATATTGATGAACGGGG	25	5
				Tcr-miR-2c-3p_L+5R+1	CGATCTCACAGCCAATATTGATGAAC	26	4
				Tcr-miR-124c-3p_R+3	TAAGGCACGCGGTGAATGCCAGAA	24	2
				Tcr-mir-219-p3	CTGCTCTCGTGTACAATTCACTGCTCTTCA	30	1
				Tcr-miR-190-5p_3ss1TA9AT13G-	AGATATGTTTGGTTACTTGGTG	22	1
				Tcr-miR-745-3p_R+1	TGCTGCCTGATAAGAGCTGTGA	22	1
				Tcr-miR-96-5p_1ss1CA	ATTGGCACTTTGGAATTGTCAC	22	1
				Tcr-miR-4988_L+1R+3	TACCTATCACACTTCAGTCCAGT	23	1
				Tcr-bantam-c-3p_R-4_1ss11AG	TGAGATCATTGTGAAAGC	18	1
				Tcr-miR-124c-3p_R+2	TAAGGCACGCGGTGAATGCCATT	23	1
				Tcr-mir-36a-p3_2ss18AC20TA	TCACCGGGTAGACATTCCTAAT	22	1
				Tcr-bantam_R+2_1ss15AC	TGAGATCGCGATTACAGCTGGTAT	24	1
				Tcr-mir-61-p5_3ss12TA19AT20CG	TGACTAGAAAGAGCACTCTGT	21	1
				Tcr-miR-124b-3p_R+1_2ss19CA20TG	TAAGGCACGCGGTGAATGAGGAA	23	1
				Tcr-miR-124a-3p_1ss18GA	TAAGGCACGCGGTGAATACTT	21	1
				Tcr-bantam-3p_R+4_1ss15AC	TGAGATCGCGATTACAGCTGGTTTTT	26	1
				Tcr-miR-7_R+3	TGGAAGACTGGTGATATGTTGTGAT	25	1
				Tcr-mir-2200-p3_2ss12AT19TC	TATTATTGTTGTGATGATC	19	1
				Tcr-mir-61-p3_3ss12TA19AT20CG	TGACTAGAAAGAGCACTCTGT	21	1

Supplementary Table S2.

Taenia solium				*Taenia crassiceps*			
miR_name	miR_seq	length	#copy of the miRNA	miR_name	miR_seq	length	#copy of the miRNA
**Tso-mir-001-3p**	**TATTGCACGTTCTTTCGCCATC**	**22**	**11414**	Tcr-mir-001-3p	TATTGCACGTTCTTTCGCCATC	22	7720
**Tso-mir-002-3p**	**GGAGATAGCGTAAAAAAGTA**	**20**	**7252**	Tcr-mir-002-3p	GGAGATAGCGTAAAAAAGTA	20	1293
Tso-mir-003-3p	TGACTAGAAAGAGCACTCACATCT	24	5317	Tcr-mir-003-3p	TGACTAGAAAGAGCACTCACATCT	24	2816
Tso-mir-004-5p	CCGATGCGGGACTCCC	16	3613	Tcr-mir-004-5p	CCGATGCGGGACTCCC	16	2273
Tso-mir-005-3p	CCGTAGCCAGACAAACT	17	2568	Tcr-mir-005-3p	CCGTAGCCAGACAAAC	16	521
Tso-mir-006-3p	TGGAATGTTGTGAAGTATGTAAA	23	1002	Tcr-mir-006-3p	TGGAATGTTGTGAAGTATGTAAA	23	397
Tso-mir-007-3p	TAAATGCAAAATATCTGGTTAT	22	713	Tcr-mir-007-3p	TAAATGCAAAATATCTGGTTATGA	24	477
Tso-mir-008-3p	GATTGCACTACCTATCGCCCATT	23	691	Tcr-mir-008-3p	GATTGCACTACCCATCGCCCATT	23	261
Tso-mir-009-3p	TATTATGCAACTTTTCACTCCT	22	535	Tcr-mir-009-3p	TATTATGCAACTTTTCACTCCT	22	553
Tso-mir-010-5p	CCAATATACTAGGAGGAGAT	20	484	Tcr-mir-010-5p	ACTAGGAGGAGATAGCGTAAT	21	63
Tso-mir-011-3p	AAAATGCACCAACTATCTGAGAAT	24	311	Tcr-mir-011-3p	AAAATGCACCAACTATCTGAGATT	24	2780
Tso-mir-012-3p	GGCTGGCTGACTGACTGACTGACT	24	270	Tcr-mir-012-3p	GCTGGCTGGCTGACTGACTGACTGACT	27	162
Tso-mir-013-3p	CTTTTTTACGCTATCTCCTCCA	22	261	Tcr-mir-013-3p	CTTTTTTACGCTATCTCCTCCA	22	40
Tso-mir-014-3p	TCACCGGGTAGACATTCCTTGC	22	245	Tcr-mir-014-3p	TCACCGGGTAGACATTCCTTGC	22	205
Tso-mir-015-3p	ACGGGCTTGGCAGAATCAGCGGGGA	25	231	Tcr-mir-015-3p	CGGGCTTGGCAGAATCAGCGGGGA	24	191
Tso-mir-016-5p	CGTGAGGCCCTTTCTTGTGCATG	23	143	Tcr-mir-016-5p	CGTGAGGCCCTTTCTTGTGCATG	23	91
Tso-mir-017-5p	TCCTACTGTCCCTATCTACTATC	23	128	Tcr-mir-017-5p	TCCCTATCTACTATCTAGC	19	47
Tso-mir-018-3p	TGAGATCGCGATTACAGCTGATA	23	126	Tcr-mir-018-3p	TGAGATCGCGATTACAGCTGAAT	23	379
Tso-mir-019-5p	GTGCGCCTGTAGCTAAGAACT	21	119	Tcr-mir-019-5p	GTGCGCCTGTAGCTAAGAACT	21	51
Tso-mir-020-3p	TTCTTGCAGAAAACGAGAGC	20	99	Tcr-mir-020-3p	TTCTTGCAGAAAACGAGAGC	20	42
Tso-mir-021-5p	AGTGGATTCGTTGCATATTATACA	24	74	Tcr-mir-021-5p	AGTGGAATCGTTGCATATTATACA	24	90
Tso-mir-022-3p	CCACTAACCAAGTAAAATACACT	23	44	Tcr-mir-022-3p	CCACTAACCAAGTAAAATACACT	23	14
Tso-mir-023-5p	GGTTGGTTGGACGGTCGGTC	20	43	Tcr-mir-023-5p	TTGGTTGGACGGTCGGTC	18	7
Tso-mir-024-5p	GGGAGAGTTGCCGAAGATGGATT	23	42	Tcr-mir-024-5p	GGGAGAGTTGCCGAAGATGGATT	23	9
Tso-mir-025-3p	TCGGGCAGGGTTGGACGCTGAGGA	24	22	Tcr-mir-025-3p	TCGGGCAGGGTTGGACGCTGAGGACATC	28	2
Tso-mir-026-5p	GTGTATTTTACTTGGTTAGTGGT	23	17	Tcr-mir-026-5p	GTGTATTTTACTTGGTTAGTGGT	23	4
Tso-mir-027-3p	GACGATCGGTCGGTCGGTC	19	12	Tcr-mir-027-3p	GACGATCGGTCGGTCGGTC	19	2
Tso-mir-028-3p	TTAGGAATCTATTGGCGAAAA	21	9	Tcr-mir-028-3p	TTAGGAATCTATTGGCGAAT	20	2
Tso-mir-029-5p	AGACCAGATTTTGCACTTTAT	21	8	Tcr-mir-029-5p	AGACCAGATTTTGCACTTTATA	22	12
Tso-mir-030-5p	TGCCCTTGTATAATGCCTTC	20	7	Tcr-mir-030-5p	TGCCCTTGTATAATGCCTTC	20	3
Tso-mir-031-3p	ATTGGCACTTTTGGAAAAA	19	7	Tcr-mir-031-3p	ATTGGCACTTTTGGAAAAA	19	4
Tso-mir-032-5p	ATTCGCCAATAGATTCCTATAT	22	6	Tcr-mir-032-5p	CATTCGCCAATAGATTCCTAAA	22	1
Tso-mir-033-5p	AAAGCAGACCTGACTTTGATA	21	6	Tcr-mir-033-5p	AAAGCAGACCTGACTTTGATA	21	2
Tso-mir-034-5p	CGGTGAAAGTTTATGCATTTACA	23	5	Tcr-mir-034-5p	CGGTGAAAGTTTATGCATTTACA	23	1
Tso-mir-035-5p	CGGCTTTTCTCGCGTTCTGAGA	22	5	Tcr-mir-035-5p	CGGCTTTTCTCGCGTTCTGATT	22	11
Tso-mir-036-3p	GTTCACAAGTGGGAAGTACT	20	5	Tcr-mir-036-3p	GTTCACAAGTGGGAAGTACT	20	2
Tso-mir-037-3p	TCAGCAGTTGTACCATTGAAAT	22	5	Tcr-mir-037-3p	TCAGCAGTTGTACCATTGAAAT	22	10
Tso-mir-038-5p	TGGCGCTTCCCAACATCACTGA	22	5	Tcr-mir-038-5p	TGGCGCTTGATTTCAACACTGA	22	19
Tso-mir-039-5p	TGGCGGTGTGCGGTGCAATTTCTG	24	4	Tcr-mir-039-5p	TGGCGGTGTGCGGTGCAATTTCTGTTT	27	27
Tso-mir-040-5p	AGCACATCGATCCACGAATC	20	3	Tcr-mir-040-5p	AGCACATCGATCCACGAATC	20	1
Tso-mir-041-3p	AAGAAGCACAACTGAGCAATA	21	2	Tcr-mir-041-3p	AAGAAGCACAACTGAGCAATT	21	1
Tso-mir-042-5p	CACTGCTGCTAGGTTGCAGCCAAAAGAGGGTG	32	2	Tcr-mir-042-5p	ACTGCTGCTAGGTTGCAGCCAAAAGAGGGTGT	32	3
Tso-mir-043-3p	CAGCATGTCGCAGTTTTCCCTAAA	24	1	Tcr-mir-043-3p	CAGCATATCGCAGTTTTCCCTA	22	2
Tso-mir-044-3p	AGGCGAGGCGATGATGATGAAA	22	1	Tcr-mir-044-3p	AGGCAAGGCGATGATGATGAAA	22	1

Supplementary Table S3.

Taenia solium				*Taenia crassiceps*			
miR_name	miR_seq	lenght	#copy of the miRNA	miR_name	miR_seq	lenght	#copy of the miRNA
Tso-5p-1041_824	AGACGTAAGTATTCGAGCAGC	21	824	Tcr-3p-181109_2	AATGTGGCTGTAACGTTGC	19	2
Tso-5p-1759_479	CGTTTGTATGGCTGCGC	17	479	Tcr-3p-860061_1	ATTGTAGGTTGAGTAAATTAATT	23	1
tso-3p-3621_210	CACTGCGAGGTATTGAGTCCTATCCCTAC	29	210	Tcr-5p-146643_3	TTTCATTGGTCGATTGCTAA	20	3
Tso-5p-5861_134	GTCAGTGTGGCCGAGCGGTCT	21	134	Tcr-3p-177806_3	TGCCCTTGTATAATGCCTTC	20	3
tso-3p-7476_102	ATCTCCTCCTAGTATATTGG	20	102	Tcr-3p-366346_1	CGTGTGAGTGAGTGAGTGAGTGGGTG	26	1
Tso-5p-7244_96	CGTGCTTCCCAATAGGCCATA	21	96	Tcr-5p-81859_6	TTTGCCCTCTAGTTACACAATCATG	25	6
Tso-5p-15867_44	TTGTGTTGCCGTGGCC	16	44	Tcr-3p-121102_4	AGTGTTGATGTCAGGTTGCTTCT	23	4
Tso-3p-16143_40	TGACTACCATCAAGAGTCCAGACTGTCTC	29	40	Tcr-5p-3735_172	GGAGTGGAGTGGAGTGGAG	19	172
Tso-3p-20970_30	TGGATGATCGGTTGGTT	17	30	Tcr-5p-267320_2	GCCGGGAGAACTCGAAAATG	20	2
Tso-5p-21844_28	TGAAGGCTATTGTCAACTAGGACCA	25	28	Tcr-5p-51323_12	AACTGAACAGCCTGTGGAATGCGTGTCTAC	30	12
Tso-5p-25867_24	AACTCAGCTATTCACACCTGCC	22	24	Tcr-3p-80608_6	GTGTCTGATGTGCGTCGATTGCTGAGT	27	6
Tso-5p-33298_20	GAGCCCCACAGCCCTGGACT	20	20	Tcr-3p-902736_1	TCACGAGCACGTGACGTGGTAGCA	24	1
Tso-3p-32594_20	TAAATGCATTTTCTGGCCCGTAATAT	26	20	Tcr-3p-269917_3	CGGCTGTGTGTGCGTGTGTG	20	3
Tso-3p-35912_16	GCACGCACGTCACAGGT	17	16	Tcr-5p-469906_1	CGGGTGCTCGAGCTGTGAT	19	1
Tso-3p-63715_11	CTAGTATTGTCAACTTACAGAA	22	11	Tcr-3p-255980_2	GATGCCGTAGCGCTCCGATA	20	2
Tso-5p-51384_11	AACAGCCTGTGGAATGC	17	11	Tcr-5p-675930_1	AGAAGGCATTATACAAGGAAA	21	1
Tso-5p-51094_11	GTAGGTTGACGATAATAGCATA	22	11	Tcr-5p-1028129_1	TGGTTTAGTAGTTCTCTGG	19	1
Tso-5p-50602_11	GACTCCCTGTTGATTCTGAT	20	11	Tcr-3p-982512_1	AATGGCTGTGTCGTTCCCT	19	1
Tso-3p-57650_10	AGGTCACGTCGGGGTCACC	19	10	Tcr-3p-217446_2	CGGTTGTGGCGAGGCGTCTCCT	22	2
Tso-5p-61725_9	GTGGGTAGAAAGTGCCTTTTACA	23	9	Tcr-5p-394106_1	ATGCCGGGTTGACGTACAAAGG	22	1
Tso-3p-74528_8	TGGGTGGGTGGGTAGTT	17	8	Tcr-3p-242925_2	TGTGACAGCGCTTTCTTCAAA	21	2
Tso-5p-71894_7	TTTTTCATTATGAGTCTGCC	20	7	Tcr-5p-101933_5	GCCCTGGTCTATCCTCATGCC	21	5
Tso-3p-75508_7	AGAAAGAGGACCCGACTTTGAT	22	7	Tcr-3p-6584_90	ACGAGCTCAACCTATTCTCAAACT	24	90
Tso-5p-75141_7	TTGGAATGTCTGCTTTCAGAGC	22	7	Tcr-5p-185627_2	TGGCATTGAATTGGAGCAAA	20	2
Tso-3p-133897_5	GCATGGTACCTGCTCATGAAA	21	5	Tcr-5p-3070_211	TCATGGACCGTAGCCAGACAAACT	24	211
Tso-3p-119385_4	AACGTTAGTGATGACGAGCT	20	4	Tcr-5p-306745_2	TACAAGGGCAGGATACATATTC	22	2
Tso-3p-122020_4	ACACTGAAATCGCCTTTGAC	20	4	Tcr-3p-1051365_1	AAACGTCAGTTTGGTGAACCGAAA	24	1
Tso-5p-160645_4	TATAGGAAATCTCGGGCCGCT	21	4	Tcr-5p-41949_15	GAAACGAGCTCAACCT	16	15
Tso-5p-117934_4	GATCTGAGGTAGTGTTTCGAATGTCT	26	4	Tcr-5p-420114_1	AAGGGGATGATGGGAGGTTTAATGCCT	27	1
Tso-3p-118835_4	GGTCCCTTGTGAATCCTGAGA	21	4	Tcr-5p-828252_2	ATCCTGAATTGTTCACATGCTGTTG	25	2
Tso-3p-159957_4	GTCCACTCTCGTGGGCGTGGGTTC	24	4	Tcr-5p-938728_1	TGTCGACTCACTGACTTATG	20	1
Tso-3p-169754_4	TGTTGTATTAACGTAAACTGAAATCGCCT	29	4	Tcr-5p-1013887_1	CGGTGGTAAGACATCAAGGACTTGG	25	1
Tso-5p-126295_4	TACCTTGTTGATCGTCGAGCT	21	4	Tcr-5p-7550_77	TCGGTTCTTATCGGGTATATCATG	24	77
Tso-3p-111640_4	TTCTTATCTATTGTAGA	17	4	Tcr-5p-758049_1	TGGCGCCTCCCAACATCACTG	21	1
Tso-5p-168456_4	TAACGTAAACTGAAATCGCCT	21	4	Tcr-5p-22224_26	ACAATACGGTCGCAGAGATT	20	26
Tso-3p-224665_3	TCAGTTAGATTTCCTCTTTCGCC	23	3	Tcr-3p-1035060_1	ACAAGTCGGTGAGTAGACAAA	21	1
Tso-3p-158797_3	TTGAGCGTCATATTGGTATGTC	22	3	Tcr-5p-56912_10	TAAGTAGAAGATTGTGGGTTCGTGTCCCACC	31	10
Tso-5p-134438_3	AGCTCATCCGTAATTATCTGGCCT	24	3	Tcr-3p-761469_2	CCGTACCCGAAACCGAC	17	2
Tso-5p-137843_3	GTAAAAGAAGGCGTGGTACT	20	3	Tcr-3p-223239_2	GTAGCGTCGTGTCTCTTGGC	20	2
Tso-5p-148919_3	TTGCGTTGGCGGGCGGGC	18	3	Tcr-5p-972081_1	AAAGAGGTTTTAGGTTCTTATGGTTCATCT	30	1
Tso-5p-139117_3	TTGTGATAGCTGACACGGGAAT	22	3	Tcr-3p-652175_1	ACTGACTGACTGACTGACTATT	22	1
Tso-5p-159824_3	AGACCTCCGTCGGTCTTTTTGCTACATC	28	3	Tcr-3p-958312_1	AGATGCCCAGCTTGAGAATCG	21	1
Tso-5p-177938_3	TGTGAGCGTGTGGTGAG	17	3	Tcr-5p-680397_1	TCAGCCTTCTGACGTTTAACC	21	1
Tso-3p-144481_3	TCGACGATCAACAAGGTAGG	20	3	Tcr-3p-317098_1	GAGTCCTGTGGCTTCTCATA	20	1
Tso-5p-275416_2	CACGCCATCCCACGGCGTACT	21	2	Tcr-5p-259696_2	TACCTGATTCTCTGGATGGCA	21	2
Tso-3p-676543_2	TCTCGAAAATGGTGGGTTGCT	21	2	Tcr-5p-578326_1	CCTGTACTGTGTAATACATCGTA	23	1
Tso-3p-190652_2	GACCATGGACATGTACAGAGAA	22	2	Tcr-3p-920033_1	CATGTGATGTGGAAGAATAGG	21	1
Tso-3p-180941_2	ATCGGTGTGAAAGAAGTGCATA	22	2	Tcr-3p-12915_42	CGAGTCCCACCGTGGGTGCCA	21	42
Tso-3p-264348_2	TTTTGGTAGATTTTTAATGGA	21	2	Tcr-5p-289116_2	AGCTCATCCGTAATTATCTGGCCT	24	2
Tso-3p-724813_2	GTTGTTCGTCAGGTTATTC	19	2	Tcr-3p-354879_1	ATTTTTCCGGTAGTCAATGC	20	1
Tso-5p-247348_2	ATCCTGAATTGTTCACATGCTGTTG	25	2	Tcr-3p-1081589_1	AATGTGAAGAATGAGGAGCT	20	1
Tso-5p-489145_2	TGTGCATGCATGTGAAGGGAAGGGGTGAG	29	2	Tcr-5p-245400_2	GTATTTGATGACATTATTCGATGGGCGGGCT	31	2
Tso-5p-202569_2	TGTTTGATTAATTTTTCTTCGAGGAT	26	2	Tcr-3p-666889_1	TGAGGAATATGTGAAA	16	1
Tso-5p-361986_2	CGACGTCCCTCAGCTGCTGCTGC	23	2	Tcr-3p-635043_1	GTCGGCATTGAGTTGGGCTACGTC	24	1
Tso-5p-186167_2	ATAGGCGGCATTTCAC	16	2	Tcr-5p-1058779_1	ACCATTCGCCAATAGATTCCTTT	23	1
Tso-5p-185012_2	CCGTGATCCGTGACATTTGGGCA	23	2	Tcr-5p-758622_1	TCCCAAGCAGGGCTGTGATACT	22	1
Tso-5p-265608_2	AGAAGATCTCCATTATTGTTAGACAAACGA	30	2	Tcr-5p-7506_77	CGTGCTTCCCAATAGGCCATA	21	77
tso-3p-253729_2	TTTGTTAAACTGACGAGCACT	21	2	Tcr-5p-139030_3	AACTCAGCTATTCACACCTGCC	22	3
Tso-5p-280902_2	AACCTATCACACTTCGGTCC	20	2	Tcr-5p-3679_171	AGGTCGGGTGGCTAGCTGGTCGATT	25	171
Tso-3p-360113_2	CGGCCGGCGCCTAGCAGC	18	2	Tcr-3p-94067_6	AGCTTCGTCTGGTCTTGCTGCA	22	6
Tso-3p-225354_2	CCTACTTGTGAATGACGTGC	20	2	Tcr-5p-577023_1	TAAGTAAAGAAACAATGATG	20	1
Tso-5p-499291_2	AAACGAATCTTTGTAACTAA	20	2	Tcr-5p-13552_41	TGGCAAGATACTGGCGAAGCT	21	41
Tso-5p-238677_2	CTGTCCTCTGCACTCCGAAGTT	22	2	Tcr-3p-790917_1	CGTGTCGGCATTGATCGCGTT	21	1
Tso-5p-195286_2	TGCATGCTTCCAGCGCAGGATAGAT	25	2	Tcr-5p-902713_1	CAATACAAAATGGTCGGAAA	20	1
Tso-3p-227446_2	GCATAGATGGCGAACAGTTCT	21	2	Tcr-5p-890294_1	TTAACGTCCATGAAGAAGTCA	21	1
Tso-3p-252880_2	TCTCAGGCATTAAATTCTATTGTT	24	2	Tcr-5p-686787_1	CGGATTGTCCGTCCGGTAACCT	22	1
Tso-5p-235083_2	TTGCGCGGCACAACATCACTGA	22	2				
Tso-3p-182073_2	CGGGGACGACGCTCGAAAGATAA	23	2				
Tso-3p-204866_2	AAGGTTTTCTGCAGGCGC	18	2				
Tso-3p-277230_2	CAGGACGGAGCCCTTTTACCAGTA	24	2				
Tso-5p-196151_2	ACGGATAAGATTTGCGTGCT	20	2				
Tso-5p-270181_2	ACTTCTCGCTATGGAGGCT	19	2				
Tso-3p-264484_2	TCATAACAGAAGGCCTGAGAA	21	2				
Tso-5p-248174_2	CTCTAGCTTGGCAGTTGGAAACACC	25	2				
Tso-5p-242104_2	ATGTGATTGTGGCACT	16	2				
Tso-5p-227952_2	CGGTGCGAGAGTTGGGCT	18	2				
Tso-5p-283211_2	GTGATTCCGTGGCGCGACT	19	2				
Tso-3p-179715_2	ACACTAGCTGTGAACCGTCT	20	2				
Tso-5p-917011_2	GCGGTAGGTAGGTAGGTAGG	20	2				
Tso-3p-201586_2	GGGACGGAAGTCTGAAAGGTTTATATT	27	2				
Tso-3p-634052_1	ACTGACTGACTGACTGACTATT	22	1				
Tso-5p-1042266_1	TGTTGCGATCTTGGCTGGTGCGGAC	25	1				
Tso-5p-1057051_1	CGCGCCCTTGTGTTCTGCAAA	21	1				
Tso-3p-631543_1	TAGTGCCATCAATTCCGCGAT	21	1				
Tso-5p-917983_1	TTGTGGACTGACTGGCTGATATT	23	1				

Supplementary Table S3.

Tso-3p-873375_1	CATTGCTCAGGTGTGCTTCT	20	1
Tso-5p-1001077_1	GCTACGGTCGATTGAGCGAATCATCGAGG	29	1
Tso-3p-892723_1	AGCTGGCACGGGAGGCTGACAA	22	1
Tso-5p-713627_1	AAAGCACTGATGGCGACTC	19	1
Tso-3p-1028603_1	CAGGCCTCGGAGACTTGAGAA	21	1
Tso-3p-685883_1	GGGAAACATCTGTGAACAAG	20	1
Tso-5p-501890_1	AACATAGAAGCTACTCAAAT	20	1
Tso-5p-803521_1	TGCGATGAAACGCAGACACT	20	1
Tso-3p-638819_1	TCAGGACCTTTGCGTGTA	18	1
Tso-3p-916550_1	CGAGTGCTGGCTCTAAAAGGC	21	1
Tso-3p-586881_1	CTTCGTCTGGTCTTGCTGCATA	22	1
Tso-3p-363269_1	TTTTAAATGTGCGCATGTATA	21	1
Tso-3p-369089_1	CGGGGTGTGGGGATGACC	18	1
Tso-5p-320798_1	TGCCACTCCCTCACTGTGACT	21	1
Tso-5p-414125_1	AACGCCAGCTTTGAGAATCGACCT	24	1
Tso-3p-401243_1	ATGGGGGCGGAACGGGTTCGGTGC	24	1
Tso-5p-721757_1	TCCCTCTTTAGTCGCGCCTCT	21	1
Tso-3p-445940_1	ACTCGGTAAATTGGTTGT	18	1
Tso-3p-1000447_1	TGGCGGCAGCTGAAGAAGTCGA	22	1
Tso-5p-369971_1	ACTTGTTCGTGAGCCTGCC	19	1
Tso-3p-627904_1	CTGTCAATAAGCATTCCAAAACT	23	1
Tso-5p-587291_1	TCGCAGATTTGAGTAGCTTCT	21	1
Tso-3p-773302_1	ATCGATCCACGAATCACTGT	20	1
Tso-3p-650687_1	CACTAGTAGAATTTATATTGA	21	1
Tso-5p-960965_1	GGCAAGATACTGGCGAAGCTGA	22	1
Tso-5p-1006879_1	CATTATACAAGGGCAGGATAAATC	24	1
Tso-3p-835421_1	GAAGTGACACTAGGCGTGAA	20	1
Tso-3p-303743_1	TGAATAACCAAGTTGATCAGT	21	1
Tso-5p-411320_1	AATGCCAGTTCGCCTAGCTTGCGGAT	26	1
Tso-3p-291570_1	CAAGAGACACGACGCTACAATA	22	1
Tso-3p-344446_1	CTGCTTGTGGCAAACTTTGGA	21	1
Tso-5p-926476_1	GCGATGGAAGTAAAATGTCTGAGAGGA	27	1
Tso-5p-768409_1	TGCGTGTGGGATAAAAA	17	1
Tso-5p-710831_1	CAGTGTCCATGTCTCAAAGAA	21	1
Tso-5p-684765_1	TAAGGACACTGTAGCCACTGG	21	1
Tso-5p-789410_1	TAGAGTGGCAACAAATAAACCTGTT	25	1
Tso-5p-378626_1	TTTAAGGTAAAAGAAGGCATTATT	24	1
Tso-5p-531974_1	GGTGGTTTCTAGCGTTGGCC	20	1
Tso-3p-824818_1	AGCTTCCAGACGTGTCGATGAACT	24	1
Tso-5p-968288_1	AAGGCATTATACAAGGGCAGAATT	24	1
Tso-3p-750143_1	TACTTCGTAATAAATAGCACT	21	1
Tso-3p-431669_1	CACCACTGAAACGCAGACGCAG	22	1
Tso-3p-940314_1	GATTGGCCAGAGTGAAACGATA	22	1
Tso-5p-833367_1	TATCTGAATGATGCGGTCT	19	1
Tso-5p-559113_1	AATGGATCGAGACACATGCAAA	22	1
Tso-5p-525784_1	TCTTGGCAGTTCGACTGGACT	21	1
Tso-3p-496967_1	ACGTCACTTCATACACAAAGGAATT	25	1
Tso-3p-374419_1	TGCAGCACACTCCTTTCCTCTGCGTCATA	29	1
Tso-3p-357668_1	TCATGATTGATGAAGCGAATGCACT	25	1
Tso-3p-781431_1	TTGCCTGGTTGAATGCGATC	20	1
Tso-5p-881335_1	GACGGATAAGTGGGCTGTCGTAGGC	25	1
Tso-3p-998827_1	AGAAATGGATTGGCCGGGCT	20	1
Tso-3p-325091_1	GGTTCCGCCGTGTAG	15	1
Tso-5p-718772_1	TAGAGGAGGCGATCGTCTACT	21	1
Tso-5p-688495_1	TTGCTGATTTCGGGTGTCTGGCTGCTTC	28	1
Tso-3p-988348_1	GCATTGGCCCACGTCGGCTTGAAATCGGTGAA	32	1
Tso-5p-1022081_1	AGCACTGGTCCGATTCCGACTGGGAAA	27	1
Tso-3p-781222_1	GCTCCCATTGGCCAGCAGTTCTGCC	25	1
Tso-3p-395893_1	ACAGCGAAATGGCAGAAGTAAT	22	1
Tso-3p-1058103_1	AGGAGCTCTTTTTGTCATCA	20	1
Tso-3p-532788_1	TGCAGGGTCCCGGTTGGTATCC	22	1
Tso-3p-865650_1	ACTAGCCATTTGGTCGGCCC	20	1
Tso-3p-905089_1	TGTCCATCACCTGCCAGGTAT	21	1
Tso-5p-410173_1	AGTCTCGGGACGCTACATTGATAT	24	1
Tso-5p-440945_1	AGTGTCCATGAGTCTGATGAGGAGAGCG	28	1
Tso-5p-775228_1	GGATGGGATTGGCTCTGGTCC	21	1
Tso-5p-327799_1	ATTGCAGGCCTTGAACACGTA	21	1
Tso-5p-846721_1	TTCATTGGTCGATTGCTAGT	20	1
Tso-3p-715835_1	TCCGCTGTGTGGATTGTGAAGGCTGCC	27	1
Tso-5p-828579_1	GACGTAGTTTACCTATCTTGA	21	1
Tso-5p-653076_1	TTGCCCCGCTTGGAGCACCAAGAA	24	1
Tso-5p-384138_1	TGGTCCAAGTTCTGTCAGATG	21	1
Tso-3p-336828_1	TGGTTCAGTAGCGATCTCTTGCC	23	1
Tso-3p-558042_1	AACGAGTAGTCTGTTTTATAATAATA	26	1
Tso-5p-535786_1	TGGAGAAAGGCATTCGGTGG	20	1
Tso-3p-427859_1	CGAGCACGTGACGTGGTAGCAGA	23	1
Tso-5p-398526_1	TGGGGACGCAGTGGGGACAGCA	22	1
Tso-3p-855995_1	TCGGTAAGATCTATGGATTATCA	23	1
Tso-5p-843422_1	TCCCTCACATCTCATAACAGAA	22	1
Tso-5p-801641_1	GTAAGTTGATAAGAGTAGTAT	21	1
Tso-3p-947026_1	TACTGGAGATACTTTCAGTCATTTTGGCGA	30	1
Tso-3p-464300_1	ACTGTCTCCACTTTGATCTCCAAA	24	1
Tso-3p-621411_1	TGCAGATCAAAATCAACAGGGAGT	24	1
Tso-3p-552747_1	CAAATGTCACAATCGGAAGT	20	1
Tso-3p-885254_1	GTATACCGTATGTTTACCA	19	1
Tso-5p-536175_1	GAGGTAGGATTTGCAACGAAGCTG	24	1
Tso-5p-559051_1	CGCCGTAGTGCAGTGAATGACACCAAGA	28	1
Tso-3p-431108_1	AGGGTGCTGAGGCTGTATTCGAGTC	25	1
Tso-5p-758828_1	TACACGTCAAAGGTGTGCTTTGCT	24	1
Tso-5p-665823_1	CAGACAGGCAGGCAGACACT	20	1

Supplementary Table S3.

Tso-3p-973987_1	ATCCCAGTCGTGGTAGTTCC	20	1
Tso-3p-799766_1	CTGGCGGAGTTGAGAAGGGGGTGC	24	1
Tso-3p-797572_1	TGGTCGTGTGGTTCCCGCCC	20	1
Tso-3p-903123_1	TCTACGGGCCTTCATATCTGT	21	1
Tso-5p-553121_1	TATTATGCTCTAGTACTATA	20	1
Tso-3p-854233_1	GGAGTTCTGACTAAGTTGAA	20	1
Tso-5p-328997_1	GGGGAGTTACTAGCAGTTCCGAACA	25	1
Tso-3p-730142_1	ATGGTGTGAGTCACAGAATGATT	23	1
Tso-3p-542497_1	TTTGTTACGAGGACCTGTC	19	1
Tso-3p-515408_1	AGGTTACTGGACTTGGATGAATGTC	25	1
Tso-5p-787434_1	CCGACGCGAACTATGGCCCT	20	1
Tso-5p-982828_1	CCCAACCCCGGAGACTTTGA	20	1
Tso-3p-700225_1	TTAGGGCAAAGGCACGAAGTC	21	1
Tso-5p-441389_1	ACACTTGAAAAATCCTGATT	20	1
Tso-3p-877353_1	AAACGGCGGACGAAAACTTGCTGT	24	1
Tso-3p-657806_1	TACGTGCGCTCTTGTAGGCTGT	22	1
Tso-3p-669440_1	TTAAACGTCAGTTTGGTGAACCT	23	1
Tso-5p-863967_1	GAACTGCTCGATTCTCGTGAA	21	1
Tso-3p-632191_1	TGGAGGAATTTGATTGAAAATC	22	1
Tso-5p-569859_1	GTTGGTTGATCGGATG	16	1
Tso-5p-661446_1	TCAGCCTTCTGACGTTTAACC	21	1
